# New arylated benzo[*h*]quinolines induce anti-cancer activity by oxidative stress-mediated DNA damage

**DOI:** 10.1038/srep38128

**Published:** 2016-12-06

**Authors:** Dharmendra K. Yadav, Reeta Rai, Naresh Kumar, Surjeet Singh, Sanjeev Misra, Praveen Sharma, Priyanka Shaw, Horacio Pérez-Sánchez, Ricardo L. Mancera, Eun Ha Choi, Mi-hyun Kim, Ramendra Pratap

**Affiliations:** 1 Department of Biochemistry, All India Institute of Medical Sciences, Jodhpur-342005, India; 2 Department of Pharmacy, College of Pharmacy, Gachon University, 155 Gaetbeol-ro, Incheon, Korea; 3 Department of Biochemistry, All India Institute of Medical Sciences, New Delhi-110029, India; 4 Plasma Bioscience Research Center, Kwangwoon University, Nowon-Gu, Seoul 139-701, Korea; 5 Department of Chemistry, University of Delhi-110007 India; 6 Computer Science Department, Catholic University of Murcia (UCAM) E30107, Murcia, Spain; 7 School of Biomedical Sciences, Curtin Health Innovation Research Institute and Curtin Institute for Computation, Curtin University: GPO Box U1987, Perth WA 6845, Australia

## Abstract

The anti-cancer activity of the benzo[*h*]quinolines was evaluated on cultured human skin cancer (G361), lung cancer (H460), breast cancer (MCF7) and colon cancer (HCT116) cell lines. The inhibitory effect of these compounds on the cell growth was determined by the MTT assay. The compounds **3e, 3f, 3h** and **3j** showed potential cytotoxicity against these human cancer cell lines. Effect of active compounds on DNA oxidation and expression of apoptosis related gene was studied. We also developed a quantitative method to measure the activity of cyclin-dependent kinases-2 (CDK2) by western blotting in the presence of active compound. In addition, molecular docking revealed that benzo[*h*]quinolines can correctly dock into the hydrophobic pocket of the targets receptor protein aromatase and CDK2, while their bioavailability/drug-likeness was predicted to be acceptable but requires future optimization. These findings reveal that benzo[*h*]quinolines act as anti-cancer agents by inducing oxidative stress-mediated DNA damage.

Cancer remains a major health problem worldwide with significant morbidity and mortality rates, causing about 14.6% of all worldwide deaths^1^. Currently, radio and chemotherapy are used for cancer treatment. In many cases these are successful, while in some cases they lead to severe compromising of the immune system due to lack of cell selectivity^2^. The treatment of cancer is indeed associated with many side effects, which include mutagenicity, tumorigenicity, skin irritation, developmental toxicity, hepatotoxicity, drug-induced cancer, alopecia etc resulting in a strong need for novel anti-cancer agents. In the last decade, several quinoline analogues have been shown to have anti-cancer activity. The quinoline ring system and its fused derivatives are significant structural units and are found in various alkaloids, therapeutics and synthetic analogues, which exhibit diverse biological activities^3^,^4^,^5^. Various quinolines have been reported as anti-malarial, anti-inflammatory, anti-asthmatic, anti-bacterial, anti-hypertensive and platelet derived growth factor receptor tyrosine kinase (PDGF-RTK)-inhibiting agents^6^. A large variety of quinolines are reported to exhibit substantial anti-cancer activity^7^,^8^,^9^,^10^,^11^,^12^,^13^,^14^ through a variety of mechanisms, such as cell cycle arrest in the G2 phase^13^, inhibition of topoisomerase^15^, inhibition of tubulin polymerization^16^ and the inhibition of tyrosine kinases^17^,^18^,^19^.

Herein, we are reporting the anti-cancer activity of ten benzo[*h*]quinoline derivatives against human skin cancer (G361), lung cancer (H460), breast cancer (MCF7) and colon cancer (HCT116) cell lines. The most potent compounds identified (**3e**, **3f, 3h** and **3j)** showed effective cytotoxicity against these cancer cell lines with IC_50_ values ranging from 4.7 to 7.6 μM. Molecular docking studies were also performed to predict the likely interactions of these molecules in the binding site of their human target receptors cyclin-dependent kinase-2 (CDK2) and aromatase. The oral bioavailability/drug-likeness of the compounds were evaluated through predictive absorption, distribution, metabolism, and excretion (ADME) screening. Oxidation of DNA (8-hydroxy-20-deoxyguanosine (8-OHdG) formation) extracted from all cancer cells was measured after exposure to active arylated benzo[*h*]quinolines. The action of these active compounds on oxidative signaling cascades was characterized by measuring the apoptosis-related mRNA gene expression of ATM (Ataxia telangiectasia mutated is a serine/threonine protein kinase)^20^, Bax (BCL2-associated X protein regulated by the tumor suppressor P53)^21^,^22^,^23^ and H2AFX (H2A histone family, member X)^24^. Apoptosis was studied by flow cytometry.

## Results and Discussion

### Chemistry

**Figure 1:**
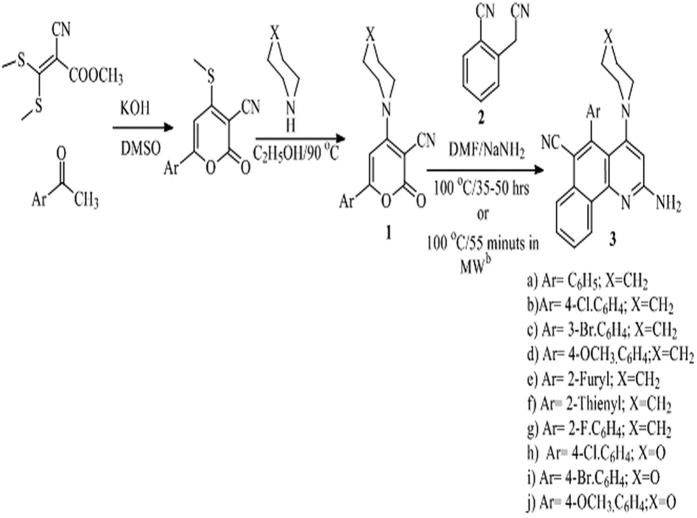
Synthesis of 6-aryl-4-*sec.*amino-2-oxo-2*H*-pyran-3-carbonitriles 1 and 2-amino-5-aryl-4-*sec*.amino-1-yl-benzo[*h*]quinoline-6-carbonitriles 3^a^.

The synthesis of benzo[*h*]quinolines is presented in Fig. 1. The required precursor was synthesized in two steps. In the first step, synthesis of 6-aryl-4-methylthio-2-oxo-2*H*-pyran-3-carbonitriles was performed by the reaction of methyl 2-cyano-3,3-bis-methylthio-acrylate with various aryl/heteroaryl methyl ketones in dimethylsulphoxide (DMSO) under basic conditions at room temperature. Further, 6-aryl-4-*sec.*amino-2-oxo-2*H*-pyran-3-carbonitriles **1** was obtained by amination^25^ of 6-aryl-4-methylthio-2-oxo-2*H*-pyran-3-carbonitriles by various secondary amines in refluxing ethanol (Fig. 1)^26^,^27^,^28^. Synthesis of highly functionalized benzo[*h*]quinolines was carried out by stirring an equimolar mixture of 6-aryl-4-*sec.*amino-2*H*-pyran-2-one-3-carbonitriles, 2-cynomethylbenzonitrile **2** and sodium amide in N,N-dimethylformamide at 100 °C for 35–50 h (Fig. 1). The presence of an electron donating or withdrawing functional group on the aryl group at the C-6 position of the pyran ring did not affect the yield of the desired product. Interestingly, the presence of thienyl and furyl rings in *lieu* of an aryl ring required longer duration of the reaction and afforded good yields. Synthesis of 2-amino-5-aryl-4-*sec.*aminobenzo[*h*]quinoline-6-carbonitriles **3** containing piperidine and morpholine at the C-4 position was also carried out. Conventional heating was replaced with microwave-assisted heating to reduce the reaction time (Fig. 1). Heating of a mixture of 6-aryl-2-oxo-4-*sec.*amino-2*H*-pyran-3-carbonitriles **1**, 2-cynomethylbenzonitrile and sodium amide in DMF at 100 °C for 55 minutes in a microwave reactor resulted excellent yields of 2-amino-5-aryl-4-*sec.*aminobenzo[*h*]quinoline-6-carbonitriles. All the studied compounds were synthesized by using the same procedure and characterized by spectroscopic analysis^29^,^30^,^31^.

### Structure activity Relationship

On the basis of result obtained after evaluation of anticancer activity, we have tried to understand the role of different functional groups on the reactivity. As it is clear from Table 1 that benzo[h]quinolines (**3e, 3f, 3h** and **3j**) are most active amongst reported compound. Compound **3f** has an IC_50_ of 5.4, 4.7, 4.9 μM against H460, MCF7 and HCT116 cells respectively, which is close to the reference compound doxorubicin (2.1 μM) and similarly compound **3e** exhibit IC_50_ value 5.3, 6.8, 7.6 and 6.8 against G361, H460, MCF7 and HCT116 cancer cell line respectively. From this result we conclude that compound with furyl group **3e** and thienyl group **3f** at position 5 of benzo[*h*]quinolines have good activity, which proposed that five member aromatic ring plays important role in activity. From the comparison of compound **3b** and **3h** it is clear that only change of piperidin to morpholine of benzo[*h*]quinolines affect the cytotoxicity in almost all of the cell lines, as shown in Table 1. The presence of the 4-methoxyphenyl group (**3j**) results in IC_50_values of 4.8, 5.2 and 6.8 μM against the H460, MCF7 and HCT116 cell lines, respectively, while the presence of the 4-bromophenyl group (**3i**) results in an IC_50_ value of 6.9 μM with the HCT116 cancer cell line. In summary, the presence of 4-chlorophenyl and 4-methoxyphenyl, furyl and theinyl ring in benzo[*h*]quinoline with suitable piperidine/morpholine moiety result in significant cytotoxicity.

### Cytotoxicity and apoptosis studies

**Figure 2:**
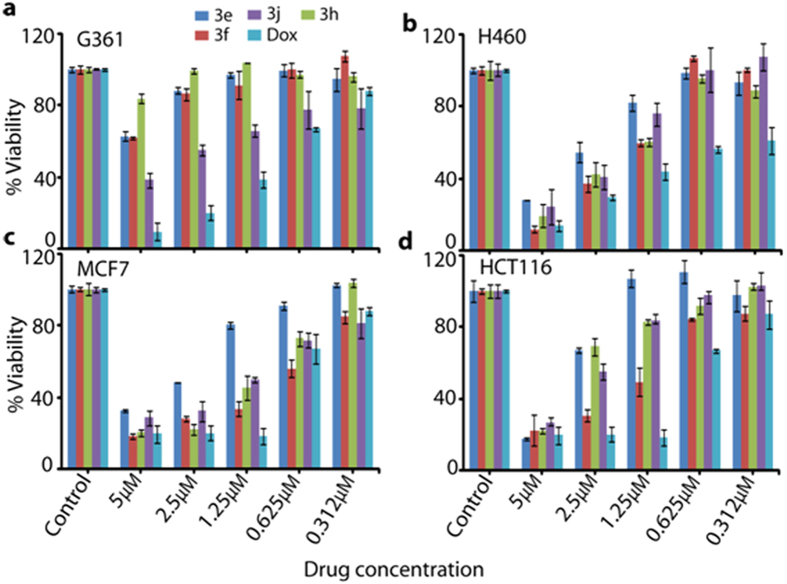
Change in cell viability upon exposure to different concentrations of compounds **3e-3f** in different cell lines: (**a**) G361; (**b**) H460 (**c**) MCF7 and (**d**) HCT116. In each experiment a negative control (no drug treatment) and a standard anticancer drug, Doxorubicin was used. All values are expressed as triplicate averages ± SD.

**Figure 3:**
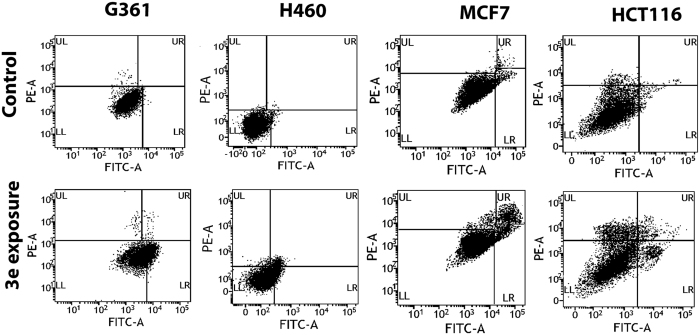
Flow cytometry analysis of the G361, H460, MCF7 and HCT116 cell lines after exposure to compound **3e** at IC_50_.

Compounds **3a–3j** was evaluated for *in vitro* cytotoxic activity by MTT assay against four human cancer cell lines: G361, H460, MCF7 and HCT116. Doxorubicin was used as a positive control (Table 1). Results obtained reveals that **3f** is cytotoxic against the HCT116 (IC_50_ 4.9 μM), MCF7 (IC_50_ 4.7 μM) and H460 (IC_50_ 5.4 μM) cell lines shown in Fig. 2a–d. Another compound, **3e** was more cytotoxic compared to doxorubicin against the G361 (IC_50_ 5.3 μM), H460 and HCT116 (IC_50_ 6.8 μM) cell lines. Compound **3h** showed significant cytotoxicity against the G361 (IC_50_ 5.5 μM), H460 (IC_50_ 5.4 μM) and MCF7 (IC_50_ 5.2 μM) cell lines but not against the HCT116 cell line. Apoptotic effect of active compound **3e** was studied at its IC_50_ concentration (see Table 1) on G361, H460, MCF7 and HCT 116 cancer cells. In this assay, cells were harvested and annex in V-FITC/PI staining was performed to determine apoptotic activity. Compound **3e** induced apoptosis in the range ~25–30% of cells in G361, H460, MCF7 and HCT116 cancer cell lines (Fig. 3). This result strongly suggests that activation of intracellular ROS and oxidative DNA damage might be responsible for induced apoptosis in these cancer cells.

### Intracellular ROS activation, mRNA expression and DNA oxidation analysis

**Figure 4:**
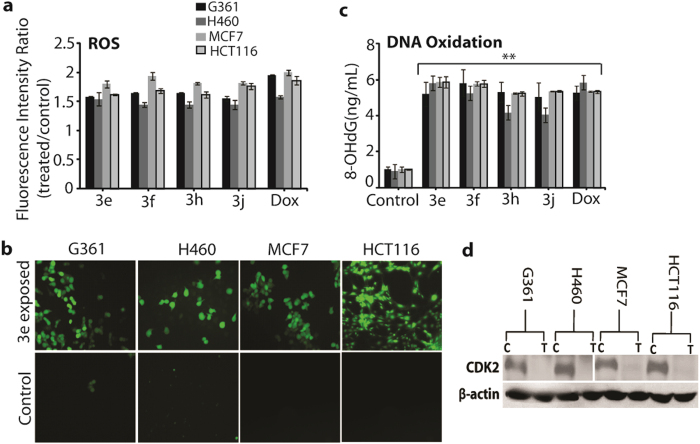
Changes in intracellular ROS and DNA oxidation. (**a**) Level of intracellular ROS in all cancer cells after exposure tocompounds **3e**, **3f**, **3h**, **3j** and Doxorubicin. All values are expressed as the fluorescence intensity ratio between compound and control. All values are expressed as triplicate averages ± SD. (**b**) Qualitative analysis of intracellular ROS level after exposure to compound **3e** in all cancer cells from fluorescence microscopy using the fluorescent probe H2DCFDA. (**c**) Amount of 8-OHdG production upon DNA oxidation upon exposure to compounds **3e**, **3f**, **3h**, **3j** and Doxorubicin. (**d**) Western blot analysis of CDK2 expression after exposure to compound **3e** in all cancer cells. All values are expressed as triplicate averages ± SD. A Student t-test was performed with respect to the control (*denotes P < 0.05 and **denotes P < 0.01).

**Figure 5:**
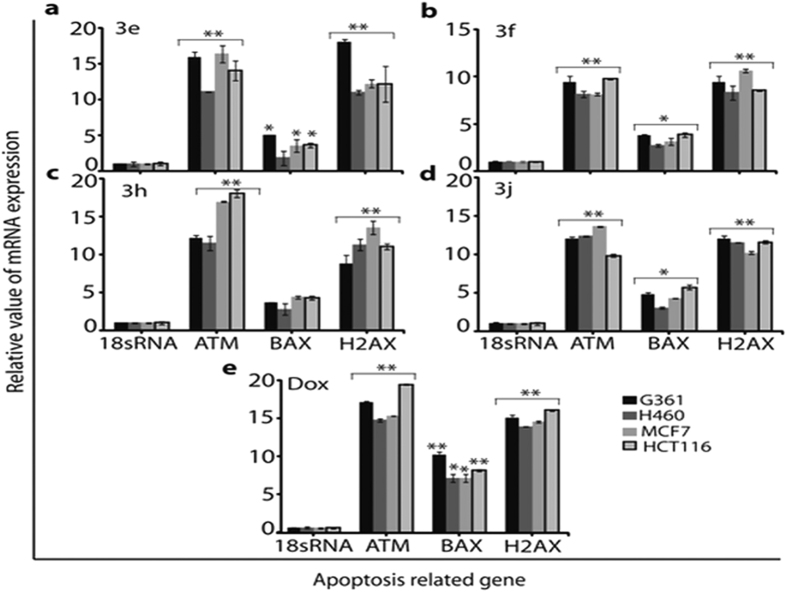
Apoptosis-related mRNA expression of H2AX, Bax, ATM, and 18 s rRNA (reference gene) after 24 h incubation with different compounds: (**a**) **3e**, (**b**) **3f**, (**c**) **3h** (**d**) **3j** and (**e**) Doxorubicin. The relative value of mRNA expression of these genes was measured by quantitative real-time PCR. All values are expressed as triplicate averages ± SD. A Student t-test was performed with respect to the control (* denotes P < 0.05 and ** denotes P < 0.01).

Intracellular ROS levels were estimated using fluorescent probes. The ROS level detected by H2DCFDA staining was dramatically increased upon addition of compounds **3e, 3f, 3h** and **3j** in all the four cancer cell lines, and was significantly higher in comparison with the positive control (Fig. 4a,b). The increase in intracellular ROS may cause oxidative stress. Hence in order to investigate the effect of the above active compounds on DNA oxidation, formation of 8-oxoguanine as a typical oxidative base lesion 7,19,29,36 was measured through the increased formation of 8-OHdG, as shown in Fig. 4c. Exposure to all active compounds lead to a substantial increase in the amount of 8-OHdG in all cancer cells. This reveals that these compounds can activate oxidative stress signaling leading to DNA oxidation. Thus, this result suggested that 3e and 3f treatment markedly induced DNA damage in cancer cells. In Fig. 4d shows that compound **3e** induce the cells death might be depends on inhibition of cyclin-dependent CDK2, a serine–threonine protein kinase associated with cell cycle progression and DNA damage. In order to establish the role of increased intracellular ROS in cancer cell death induced by the above active compounds, mRNA expression was evaluated for three oxidative stress-related genes, ATM, H2AX, and BAX (Fig. 5a–e). The ataxia telangiectasia mutated (ATM) is a serine/threonine protein kinase and responsible for activating cellular responses to DNA damage, which may be activated by H2AX and CDK2 in the DNA damage checkpoint^32^,^33^,^34^ but a mechanistic link between these two pathways has not been clearly elucidated. H2AX plays a key role in DNA damage response and is required for the assembly of DNA repair proteins at sites containing damaged chromatin as well as for activation of checkpoint proteins, which arrest the cell cycle progression^35^.

This study also demonstrates that exposure to active arylated benzo[*h*]quinolines increases levels of intracellular ROS, which may be partially mediated through changes in mitochondrial potential leading to cell death induced by H2AX and ATM over-expression. As shown in Fig. 5a–e reveals that cancer cells exposed to active compounds showed over expression of H2AX and ATM genes leading to DNA damage in cancer cells. Therefore, over expression of these genes plays a major role in initiating cell death in cancer cells through the DNA damage signalling pathway.

### Molecular modeling

The synthesized benzo[*h*]quinolines were evaluated for their inhibitory effects in different cancer cell lines. Molecular docking studies were carried out to ascertain the mode of action towards the molecular targets, that is known human breast cancer target receptor aromatase (PDB ID: 3EQM)^37^,^38^ and colon cancer target protein CDK2 (PDB ID: 2R3J)^39^,^40^. Binding affinities were predicted by the Sybyl docking total score upon docking with the Surflex-Dock program (Sybyl X 2.0). Docking studies were carried out to evaluate the binding affinity and interactions with their target proteins. Hydrogen bonds (H-bonds, with a donor-receptor distance of 3 Å) between the ligand and amino acids in the binding site of the protein were used for the ranking of compounds. The mode of interaction of the co-crystallized ligand (3-bromo-5-phenyl-N-(pyridin-3-ylmethyl) pyrazolo [1,5- a]pyrimidin-7-amine) (SCJ) within the crystal structure of CDK2 was used as a reference binding model. The binding site of CDK2 contains the important residue Leu-83 is important for hydrophobic interactions with the ligand.

**Figure 6:**
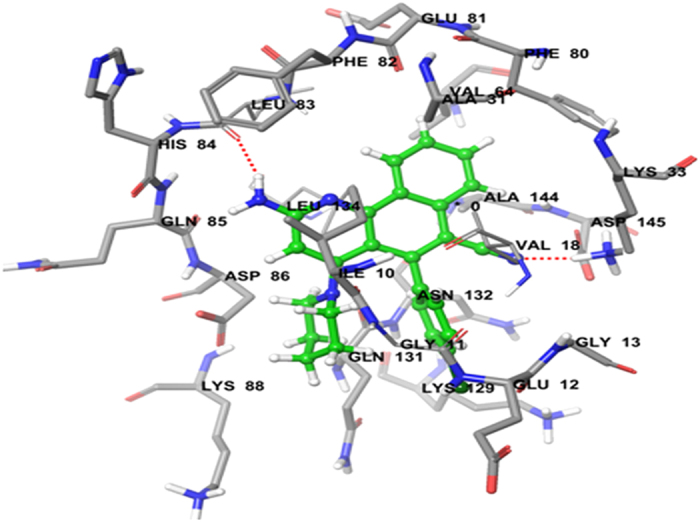
Binding interactions of compound 3e upon docking onto cyclin-dependent kinase-2 (PDB ID: 2R3J). A top docking energy (total score) of 5.4981 was predicted. Formation of two H-bonds of length 1.9 and 2.1 Å to residues Leu-86 and His-84, respectively, in the binding site were predicted.

**Figure 7:**
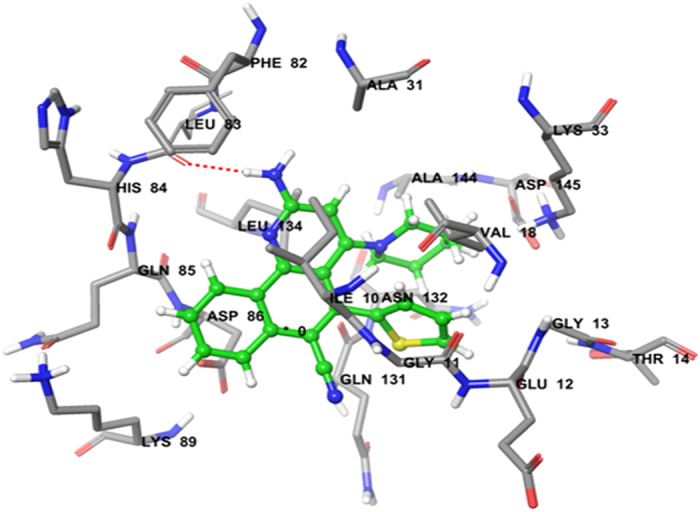
Binding interactions of compound 3f upon docking onto cyclin-dependent kinase-2 (PDB ID: 2R3J). A top docking energy (total score) of 5.5206 was predicted. Formation of a H-bond of length 2.1 Å to residue Leu-83in the binding site was predicted.

Doxorubicin (standard compound) was docked into CDK2 with a predicted total score of 5.1647. The hydrophobic cavity made by residues Asp-86, Lys-89 and Leu-83 interacted with the ligand through formation of H-bonds. Docking of compound **3e** with CDK2 resulted in a predicted high binding affinity with a total score of 5.8273 and the formation of a strong H-bond (of length 2.0 Å) to the hydrophobic residue Leu-83 (Fig. 6). The predicted binding mode of compound **3e** with CDK2 involved a number of binding site residues within a radius of 3 Å with diverse properties (Table 2):basic (polar, hydrophobic, positively charged), for example Lys-33, Lys-89, His-84; aromatic (hydrophobic), for example, Phe-82; acidic (polar, negatively charged), for example, Glu-12, Asp-145 and Asp-86; polar amide, for example, Gln-85, Gln-131 and Asn-132; hydrophobic, for example, Ala-177, Ala-131, Ala-144, Gly-13, Leu-83, Leu-134, Ile-10, Val-18 and Gly-11. As a consequence, compound **3e** was predicted to have strong hydrophobic interactions with CDK2, which provides a molecular rational for the activity in this compound (Fig. 6). Docking of compound **3f** also predicted high binding affinity with a total score of 5.5206 and the formation of a strong H-bond (length 2.1 Å) to the hydrophobic residue Leu-83, with interactions with almost the same amino acid residues in the binding site (Fig. 7).

**Figure 8:**
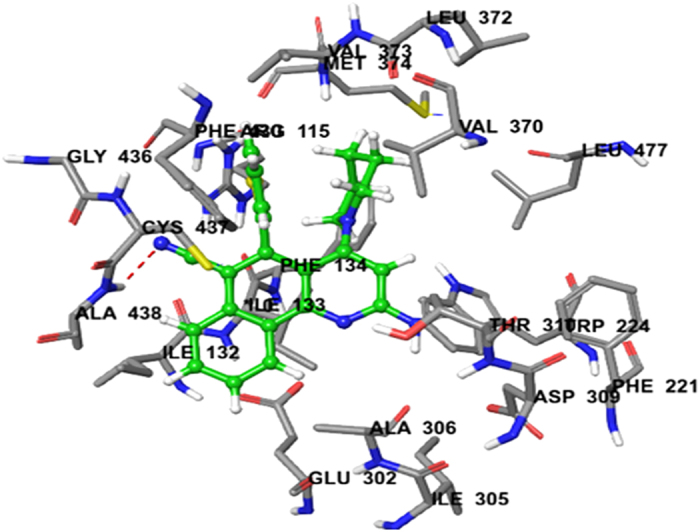
Binding interactions of Compound 3f upon docking onto breast cancer receptor aromatase (PDB ID: 3EQM). A top docking energy (total score) of 5.4971 was predicted. A H-bond of length 1.9 Å to residue Ala-438 in the binding side was predicted to be formed.

Docking of compounds **3f, 3g, 3h** and **3j** with the breast cancer target protein aromatase (PDB ID:3EQM) was also carried out. For comparison, the docking score of the control anti-cancer drug doxorubicin was predicted to be 2.2253. Docking of compound **3 f** resulted in a predicted high binding affinity with a total score of 5.4971 and the formation of an H-bond with Ala-438 (Table 3). The predicted binding mode of compound **3f** with aromatase involved a number of binding site residues within a radius of 3 Å with diverse properties: hydrophobic, for example Met-374, Ala-438, Ala-306, Ile-133, Ile-132, Leu-372, Val-373, and Val-370; aromatic (hydrophobic), for example Phe-134; nucleophilic (polar, hydrophobic), for example Cys-437; nucleophilic (polar, hydrophobic), for example Thr-310, Cys-437; polar amide, for example Gln-86, Asn-105; hydrophobic positively charged, for example Arg-115; acidic (polar, negatively charged), for example, Asp-309. As a consequence, compound **3f** was predicted to have strong hydrophobic interactions with aromatase, which provides a molecular rationale for its observed activity (Fig. 8).

**Figure 9:**
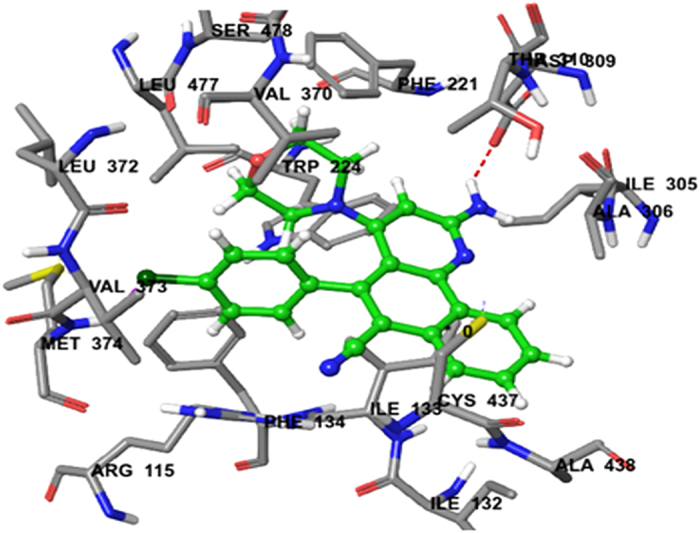
Binding interactions of compound 3h upon docking onto breast cancer receptor aromatase (PDB ID: 3EQM). A top docking energy (total score) of 2.6056 was predicted. Formation of a H-bond of length 2.0 Å to residue Asp-309 in the binding site was predicted.

**Figure 10:**
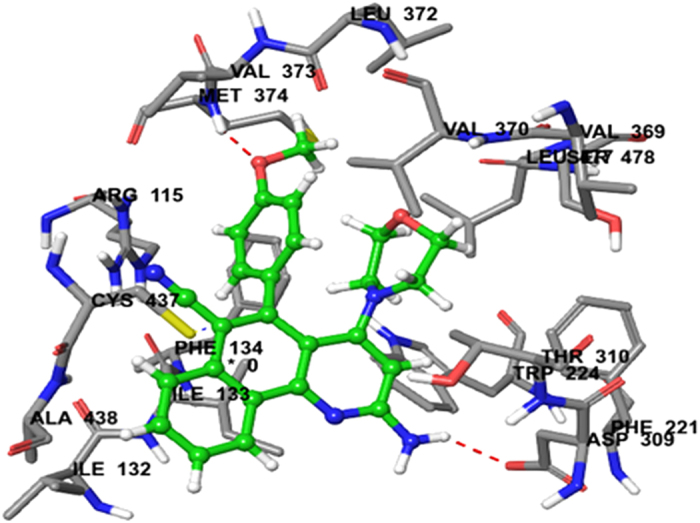
Binding interactions of compound 3j upon docking onto breast cancer receptor aromatase (PDB ID: 3EQM). A top docking energy (total score) of 5.0609 was predicted. Formation of two H-bonds of length 2.0 and 2.1 Å to the residues Asp-309 and Met-374, respectively, in the binding site were predicted.

Similarly, docking of compound **3h** resulted in a predicted high binding affinity with a total score of 5.5392. The predicted binding mode of compound **3h** with aromatase involved a number of binding site residues within a radius of 4 Å that were acidic (polar, negative charged), for example Asp-309. Compound **3 h** was also predicted to have strong hydrophobic interactions with aromatase, which explain its observed activity (Fig. 9). Likewise, docking of compound **3j** against aromatase gave a predicted high binding affinity with a total score of 5.0609 (Table 3). The predicted binding mode of compound **3j** involved several binding site residues within a radius of 4 Å that were acidic (polar, negative charged), for example Asp-309, and hydrophobic, for example Met-374. The strong hydrophobic interactions involved in the binding of compound **3j** explain its observed activity (Fig. 10).

### Pharmacokinetic and toxicity properties

Pharmacokinetic (PK) ADME properties are important descriptors for human therapeutic use of any drug molecule. These ADME descriptors were calculated for all active molecules and compared with standard ranges. All the derivatives possessed a good number of hydrogen bond donors and acceptors^39^,^40^. These derivatives were designed to increase the binding of the drug with the receptor through hydrogen bonding. These derivatives were found to follow Lipinski’s rule of 5, affording drug likeness to the designed compounds. Polar surface area was calculated to estimate the ability of the compounds to permeate cell membranes. Lipophilicity (ratio of octanol solubility to water solubility) measured through logP, which has been implicated in blood brain barrier penetration and permeability prediction. Excretion of drugs depends on MW and logP^41^.

While the benzo[*h*]quinolines showed significant anti-cancer activity, they also exhibited some PK limitations. The calculated TPSA values of these compounds were within acceptable limits. The distribution of compounds in the human body was described by the predicted blood–brain barrier coefficient (logBB), apparent Caco-2 permeability, log Kp for skin permeability, volume of distribution and plasma protein binding (log Khsa for serum protein binding)^42^,^43^,^44^. All compounds show poor aqueous solubility (Table 1 in Supplementary Information). All compounds except **3g** were predicted to be CYP2D6 non-inhibitors, and all derivatives were predicted to be hepatotoxic and highly-bound to plasma binding protein. Compounds **3c, 3d, 3i** and **3j** show medium apparent MDCK permeability. The calculated BBB values of the analogues were within the acceptable interval. The calculated values for these ADME parameters showed close similarity between the analogues and that of the reference drug doxorubicin and lie within the standard range of values exhibited by 95% of all known drugs shown in Table 4 and supplementary Figure S1.

### Toxicity risks assessment

The reproductive/developmental toxicity, Ames mutagenicity, skin irritant effects, Ames score, rat oral LD_50_ (mg/kg), and rat carcinogenic potency TD_50_ (mg/kg) were predicted for all benzo[*h*]quinoline derivatives. These compounds were estimated to possess no risk of skin irritation. Compounds **3c, 3d, 3i** and **3j** may show developmental toxicity. All compounds were predicted to possess high mutagenicity potential at high doses or long term therapeutic use in human, except for compound **3c,** which was predicted to be non-mutagenic^45^,^46^. Other predicted toxicity parameters are summarized in Table 5. The toxicity risks for benzo[*h*]quinoline derivative scanned to find the moderate to good compared with doxorubicin. Similarly, toxicity screening for USFDA rodent carcinogenicity, Ames mutagenicity, developmental toxicity potential, and carcinogenic effect irritancy are predicted to have a positive response for these benzo[*h*]quinoline derivatives.

## Material Methods

### Chemical synthesis

#### General experimental procedures

All the reagents and solvents used in this study were purchased from Sigma Aldrich and Alfa Aesar. All compounds were synthesized by the method described by Singh *et. al*.^20^ (Detail data available in S1). Detailed structural characterization is mentioned therein. These derivatives were defined as benzo[*h*]quinoline anti-cancer derivatives. In this study, we have further explored their cytotoxic effect on G361, H460, MCF7 and HCT116 cancer cell lines using *in-vitro*, and *in-silico* bioassays.

#### Reagents and consumables

G361, H460, MCF7 and HCT116 cancer cell lines were procured from KCLB (Korean Cell Line Bank, South Korea). All the cells were maintained in RPMI and DMEM supplemented with 10% fetal bovine serum, 1% non-essential amino acids, 1% L-glutamine (300 μg/mL), 1% penicillin (100 IU/ml) and streptomycin (100 mg/ml) (Hyclone, USA)^32^,^36^,^47^. Cultured cells were grown at 37 °C, 95% relative humidity and 5% CO_2_ and passaged twice a week. Cells were allowed to grow in 75 cm^2^ tissue culture flasks until confluence and then sub-cultured for experimentation.

#### Cell viability assays by MTT

Cell viability was measured by the MTT assay^34^,^36^,^48^. Briefly, 2 × 10^5^ cells/well were seeded in 96-well plates and incubated for 12 h at 37 °C in a 5% CO2 atmosphere to allow cell adhesion. Stock solutions of the compounds made in DMSO were sterilized through 0.45 micro size filter (Sartorus Stedim, biotech, USA), then further diluted to 0.2 mg/ml in incomplete medium for treatment against G361, H460, MCF7 and HCT116 cancer cells lines. A 100 μL solution of compound (**3e, 3f, 3h** and **3j**) was added respectively to a 100-μL of fresh medium in wells to give final concentrations of 5–0.312 μM/mL after serial dilution. A negative control (without drug), a positive control with anticancer drug doxorubicin was included in each assay^34^,^36^.

#### Intracellular reactive oxygen species analysis

Total ROS content inside the cells was determined using the H2DCFDA reagent. A total of 2 × 10^5^ cells/ml of G361, H460, MCF7 and HCT116 cancer cells in DMEM was incubated at the IC_50_ concentrations of the active compounds **3e, 3f, 3h, 3j** and the positive control doxorubicin respectively and then transferred to a micro-centrifuge tube. The cells were washed with PBS and 500 μl of 10 mM H2DCFDA was added. After incubation at 30 °C for 1 h, the cells were washed twice with PBS. The cells were then recovered in PBS at 30 °C for 30 min and read at 495/515 (ex./em.) nm using a microplate reader^34^,^36^,^47^. The ratio of fluorescence intensity (FI = fluorescent value at excitation wavelength of 485 nm and an emission at 535 nm of treated cells/control) was calculated between exposure and control (no treated) cells.

#### RNA extraction for quantitative real time PCR

We measured mRNA expression of three apoptosis related genes, including H2AX, ATM, and BAX. The total cellular RNA was isolated from all four cancer cells line after 24 h incubated at the IC_50_ concentration of **3e, 3f, 3h, 3j** and Doxorubicin respectively. Primer design, cDNA synthesis and quantification of every gene was carried out as previously reported^34^,^36^. Quantitative real-time PCR was done by using following forward and reverse primer sequences, as listed below. 18sRNA was amplified to ensure cDNA integrity and to normalize expression.

Primer sequences used for mRNA expression.


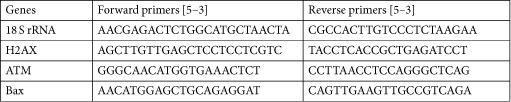


#### Apoptosis analysis by annexin V-FITC/PI staining

Cancer cells were exposed with IC_50_ concentration of active compound **3e** to detect apoptosis using the annexin V-FITC apoptosis detection kit. Treated/untreated cells were trypsinized after incubation for 12 h, after first washing with 1 ml of cold 1× biding buffer. Annexin V-FITC (0.5 mg ml^−1^) was added to each sample. After incubation for 15 min at room temperature, the cells were again washed with PBS and stained with 0.3 mg ml^−1^ of PI (Propidium Iodide) and analyzed by flow cytometry (BD FACS Verse, BD Biosciences)^34^,^36^,^47^.

#### DNA damage analysis

Genomic DNA was extracted from all four cell lines after treatment with IC_50_ concentrations of **3e, 3f, 3h, 3j** and Doxorubicin respectively, for OH-dG detection. Genomic DNA was extracted from treated/untreated cells following a standard molecular biology protocol and re-suspended in 50 μl water^34^,^36^. The same amount of genomic DNA (2 mg) extracted from cells was used for the detection of 8-OH-dG level following the standard protocol of anoxidative DNA damage ELISA kit (Cell Biolabs, Inc. USA)^34^,^36^.

#### Western blot analysis

All cells were exposed with IC_50_ concentrations of 3e compounds. After exposure, protein extraction of the cells was performed. The whole cell protein extracts from treated/untreated cells were lysed in a RIPA buffer (Cell Signaling Technology, USA) and the extracted proteins were subjected to electrophoresis in 12% SDS-PAGE and blotted onto nitrocellulose membranes^36^,^47^,^48^,^49^. The membrane was probed with the CDK2 antibodies for protein expression, (Cell Signaling Technology, USA)^50^,^51^,^52^. The bands were detected using the Super Signal West Pico Chemiluminescent substrate (Pierce, Rockford, IL, USA) and images was taken using a Vilver imaging system (Vilver, Upland, CA, USA).

#### Molecular docking study

Molecular modeling studies of benzo[*h*]quinoline derivatives were carried out using molecular modeling software Sybyl-X 2.0, (Tripos International, St. Louis, Missouri, 63144, USA). Drawing of structures and simple geometry optimization were performed with Chem Bio-Office suite Ultra v12.0 (2012) (Cambridge Soft Corp., UK). Docking of all compounds was carried out on the human anticancer targets aromatase (PDB ID: 3EQM)^37^,^38^ and CDK2 (PDB ID: 2R3J)^39^,^40^. The Surflexdoc module in Sybyl was used to construct a 3D model of the structures^53^,^54^. Details data point available in S1 of supplementary material.

#### Screening through *in silico* pharmacokinetic parameters

Pharmacokinetic (PK) properties depend on chemical descriptors of drugs, which determine their absorption, distribution, metabolism, excretion, and toxicity (ADMET) properties, which are the key descriptors for the human therapeutic use of any compound. These PK parameters were calculated using the ADMET modules in Discovery Studio v3.5 software (Accelrys, USA), and are reported in supplementary Table S1 of the Supplementary Information. Predictive mathematical ADMET models were derived with different PK parameters, namely aqueous solubility, blood-brain barrier penetration, cytochrome P-450 2D6 inhibition, hepatotoxicity, human intestinal absorption and plasma protein binding. Predictions from these models were used to quantitatively predict properties of a set contrasted with of known rules that specify for appropriate ADMET characteristics of the chemical structure of all benzo[*h*]quinoline derivatives.

#### Statistical analysis

All values are presented as the mean ± SD of the indicated number of replicates. Statistical analyses of the data were performed using Student’s t-test, and significant differences were based on P < 0.05 or P < 0.01^34^,^36^,^48^.

## Additional Information

**How to cite this article**: Yadav, D. K. *et al*. New arylated benzo[*h*]quinolines induce anti-cancer activity by Oxidative stress-mediated DNA damage. *Sci. Rep.* **6**, 38128; doi: 10.1038/srep38128 (2016).

**Publisher's note:** Springer Nature remains neutral with regard to jurisdictional claims in published maps and institutional affiliations.

## Supplementary Material

Supplementary Information

## Figures and Tables

**Table 1:** IC_50_ values of benzo[*h*]quinolines(**3a-3j)** for the growth of G361, H460, MCF7 and HCT116 cells.

Compound	IC_50_ (μM)			
	G361	H460	MCF7	HCT116
3a	42.3 (±0.1)	9.5 (±0.03)	7.5 (±1.3)	9.6 (±0.9)
3b	34.8 (±1.1)	8.2 (±2.0)	11 (±2)	7.6 (±1.1)
3c	29.2 (±1.1)	8.4 (±0.2)	10.9 (±0.7)	10.7 (±1)
3d	7.4 (±0.2)	7.0 (±1.2)	(9.7±0.7)	7.2 (±1.6)
3e	5.3 (±1.7)	6.8 (±0.7)	7.6 (±1.2)	6.8 (±0.2)
3f	7.4 (±0.5)	5.4 (±0.6)	4.7 (±0.9)	4.9 (±0.2)
3g	14.5 (±2.4)	8.2 (±1.0)	6.8 (±1.5)	9.2 (±1.2)
3h	5.5 (±0.03)	5.4 (±0.4)	5.2 (±1)	7.2 (±0.2)
3i	19.1 (±0.05)	9.5 (±0.1)	8.9 (±0.4)	6.9 (±0.1)/
3j	7.4 (±0.3)	4.8 (±0.3)	5.2 (±1.2)	6.8 (±0.5)
**Doxorubicin**	**2.1** (**±0.4)**	**1.5** (**±0.05)**	**2.4 (±0.8)**	**2.1 (±0.5)**

**Table 2:** Comparison of predicted binding affinities of active benzo[*h*]quinoline derivatives and Doxorubicin against cyclin-dependent kinase-2.

Compound	Total docking score*	Amino acids in the binding site within 3.0 Å of ligand (H-bonding residues shown in bold)	H-bond length Ă	No. of H-bonds
**3e**	5.8273	Ile-10, Gly-11, Glu-12, Gly-13, Val-18, Ala-31, Lys-33, Phe-82, **Leu-83**, His-84, Gln-85, Asp-86, Lys-89, Gln-131, Asn-132, Leu-134, Ala-144, Asp-145	2.0	1
**3f**	5.5206	Ile-10, Gly-11, Glu-12, Gly-13, Val-18, Ala-31, Lys-33, Phe-82, **Leu-83**, Gln-85, Asp-86, Lys-89, Gln-131, Asn-132, Leu-134, Ala-144, Asp-145	2.1	1
**3i**	5.1238	Ile-10, Gly-11, Val-18, Ala-31, Phe-82, **Leu-83**, His-84, Asp-86, Lys-89, Gln-131, Asn-132, Leu-134	1.9	1
**3h**	5.4981	Ile-10, Gly-11, Val-18, Ala-31, Phe-82, **Leu-83**, **His-84**, Asp-86, Lys-89, Gln-131, Leu-134	1.9 2.1	2
Doxorubicin	5.1647	Ile-10, Val-18, Ala-31, Val-64, Phe-80, Phe-82, **Leu-83**, His-84, Gln-85, **Asp-86**, Lys-88, **Lys-89**, Gln-131, Leu-134, Ala-144	1.9 2.0 2.1	3

^*^Surflex-Dock scores (total scores) are expressed in -log10(*K*_*d*_)^2^ units to represent binding affinities.

**Table 3:** Comparison of predicted binding affinities of active benzo[*h*]quinoline derivatives and Doxorubicin against breast cancer receptor aromatase.

Compound	Total docking score*	Amino acidsin the binding site within 3.0 Å of ligand (H-bonding residues shown in bold)	H-bond length Ă	No. of H-bonds
3f	5.4971	Arg-115, Ile-132, Ile-133, Phe-134, Trp-224, Ala-306, Asp-309, Thr-310, Val-370, Leu-372, Val-373, Met-374, Cys-437, **Ala-438**	1.9	1
3g	5.2132	Arg-115, Ile-132, Ile-133, Phe-134, Phe–221, Trp-224, Ila-305, Ala-306, Asp-309, Val-370, Leu-372, Val-373, Met-374, Cys-437, Ala-438, Leu-477	—	—
3h	2.6056	Arg-115, Ile-133, Phe-134, Trp-224, Ile-305, Ala-306, **Asp-309**, Thr-310, Val-370, Leu-372, Val-373, Met-374, Cys-437, Ala-438, Leu-477, Ser-478	2.0	1
3j	5.0609	Arg-115, Ile-133, Phe-134, Trp-224, Ile-305, Ala-306, **Asp-309**, Thr-310, Val-370, Leu-372, Val-373, **Met-374**, Cys-437, Ala-438, Leu-477, Ser-478	2.1 2.0	2
Doxorubicin	2.2253	Arg-115, Ile-132, Ile-133, Phe-134, Trp-224, Ala-306, Thr-310, Met-311, Ser-314, Val-370, Val-373, Phe-430, Cys-437, Ala-438	—	—

^*^Surflex-Dock scores (total scores) are expressed in -log10(*K*_*d*_)^2^ units to represent binding affinities.

**Table 4:** Predicted ADME parameters of benzo[*h*]quinoline derivatives.

Compound	Aqueous solubility	CYP2D6 binding	Hepatotoxicity	BBB penetration	Plasma protein binding
3a	1 (poor)	False (non-inhibitor)	True (toxic)	0 (Good)	True (highly bound)
3b	1 (poor))	False (non-inhibitor)	True (toxic)	0 (Good)	True (highly bound)
3c	0 (poor)	False (non-inhibitor)	True (toxic)	0 (Good)	True (highly bound)
3d	1 (poor)	False (non-inhibitor)	True (toxic)	0 (Good)	True (highly bound)
3e	1 (poor)	False (non-inhibitor)	True (toxic)	0 (Good)	True (highly bound)
3f	1 (poor)	False (non-inhibitor)	True (toxic)	0 (Good)	True (highly bound)
3g	1 (poor)	True (inhibitor)	True (toxic)	0 (Good)	True (highly bound)
3h	1 (poor)	False (non-inhibitor)	True (toxic)	0 (Good)	True (highly bound)
3i	1 (poor)	False (non-inhibitor)	True (toxic)	0 (Good)	True (highly bound)
3j	1 (poor)	False (non-inhibitor)	True (toxic)	0 (Good)	True (highly bound)
Doxorubicin	2 (low)	False (non-inhibitor)	True (toxic)	0 (Good)	False (low bound)

Cytochrome (CYP-2D6) binding, hepatotoxicity and plasma-protein binding predictions using Discovery Studio 4.0 (Biovia).

**Table 5:** Predicted toxicity risk parameters of benzo[*h*]quinolines.

Compound	Reproductive/developmental toxicity	Ames test	TOPKAT skin irritancy	TOPKAT Ames score	TOPKAT rat oral LD_50_ (mg/kg)	TOPKAT rat carcinogenic potency TD_50_ (mg/kg)
3a	Non-Toxic	Mutagen	Non-Irritant	4.52304	0.193812	3.84699
3b	Non-Toxic	Mutagen	Non-Irritant	0.925525	0.229992	1.03367
3c	Toxic	Non-Mutagen	Non-Irritant	−0.762268	0.236661	1.06625
3d	Toxic	Mutagen	Non-Irritant	3.26021	0.159558	0.531709
3e	Non-Toxic	Mutagen	Non-Irritant	4.41803	0.197063	0.8887
3f	Non-Toxic	Mutagen	Non-Irritant	3.89661	0.184627	1.06061
3g	Non-Toxic	Mutagen	Non-Irritant	1.38151	0.131322	1.14982
3h	Non-Toxic	Mutagen	Non-Irritant	2.43415	0.377348	0.472136
3i	Toxic	Mutagen	Non-Irritant	0.450189	0.51522	0.486793
3j	Toxic	Mutagen	Non-Irritant	4.8735	0.281606	0.242827
Doxorubicin	Toxic	Mutagen	Non-Irritant	23.0401	0.192388	0.756873
