# Supplementary material for: New arylated benzo[h]quinolines induce anti-cancer activity by oxidative stress-mediated DNA damage
Source: Sci Rep. 2016 Dec 6;6:38128. doi: 10.1038/srep38128 (PMC5138627; doi:10.1038/srep38128)

**Supplementary Material**

**New arylated benzo[*h*]quinolines induces anti-cancer activity by oxidative stress-mediated DNA damage**

Dharmendra K. Yadav^1,6*#^, Reeta Rai^2#^, Naresh Kumar^3#^, Surjeet Singh^4^, Sanjeev Misra^1^, Praveen Sharma^1^, Priyanka Shaw^3^, Horacio Pérez-Sánchez^5^, Ricardo L. Mancera^6^, Eun Ha Choi^3^, Mi-hyun Kim^6^, Ramendra Pratap^4*^

^1^Department of Biochemistry, All India Institute of Medical Sciences, New Delhi-110029, India

^2^Department of Biochemistry, All India Institute of Medical sciences, Jodhpur-342005, India

^3^Plasma Bioscience Research Center, Kwangwoon University, Nowon-Gu, Seoul 139-701, Korea

^4^Department of Chemistry, University of Delhi-110007, India

^5^Computer Science Department, Catholic University of Murcia (UCAM) E30107, Murcia, Spain

^6^Department of Pharmacy, College of Pharmacy, Gachon University, 155 Gaetbeol-ro, Incheon, Korea

^6^School of Biomedical Sciences, Curtin Health Innovation Research Institute and Curtin Institute for Computation, Curtin University, Perth, Australia

**Material Methods**

***Chemical synthesis***

**General experimental procedures.** All reagents and solvents used in this study were purchased from Sigma Aldrich and Alfa Aesar. All compounds were synthesized by the method described by Singh et. al.^20^. Detailed structural characterization is mentioned therein. These analogues were defined as anti-cancer benzo[*h*]quinoline analogues. The cytotoxicity of these compounds was investigated in four cancer cell lines (G361, H460, MCF7 and HCT116)using *in vitro* bioassays and *insilico* tools.

***General protocol for the synthesis of 2-amino-5-aryl-4-sec.amino-1-yl-benzo[h]quinoline-6-carbonitriles 3a to 3j***: In a vacuum dried round bottom flask a mixture of 6-aryl-2-oxo-4-sec.amino-1-yl-2H-pyran-3-carbonitrile (0.5 mmol) and 2-cynomethyl-benzonitrile (0.5 mmol; 71.0 mg) were stirred at 100^o^C in dry DMSO (4.0 mL) using NaNH_2_ (1.0 mmol; 39.0 mg) as a base for 35-50h. Completion of the reaction was monitored by TLC. The reaction mixture was poured onto crushed ice and neutralized with10% HCl before filtering the obtained solid material. Each compound was purified by silica gel column chromatography using 30% ethyl acetate in hexane. All the synthesized compounds, were characterized by spectroscopic analysis, as reported earlier.

***General protocol for the microwave-assisted synthesis of 2-amino-5-aryl-4-piperidine-1-yl-benzo[h]quinoline-6-carbonitriles 3.*** A mixture of 2-oxo-6-aryl-4-piperidin-1-yl-2*H*-pyran-3-carbonitrile (1.0 mmol) and 2-cynomethyl-benzonitrile (1.0 mmol; 142.0 mg) was transferred to a microwave vial. This was followed by addition of NaNH_2_ (2.0 mmol; 78.0 mg) and 2.0 mL dry DMF. The reactants were heated at 100^o^C under microwave irradiation for 55 min. Subsequent work-up and purification as above afforded final product.

**2-Amino-5-phenyl-4-piperidin-1-yl-benzo[*h*]quinoline-6-carbonitrile 3a:** Yield: 75% (142.0 mg);0.28 R_f_ (30% ethyl acetate-hexane), orange solid, mp: 140-142 ^o^C; IR (KBr): 3338, 3050, 2941, 2854, 2212 cm^-1^; ^1^H NMR ( 400 MH_Z_ , CDCl_3_): δ 0.58-0.71 (m, 2H, -CH_2_-), 1.20-1.41 (m, 4H, -CH_2_-), 2.32-2.42 (m, 2H, -CH_2_-), 2.83-2.94 (m, 2H, -CH_2_-), 4.88 (s, 2H, -NH_2_), 6.28 (s, 1H, ArH), 7.37-7.44 (m, 3H, ArH), 7.47-7.53 (m, 2H, ArH), 7.62-7.74 (m, 2H, ArH), 8.23 (d, *J* = 8.0 Hz,1H, ArH), 9.08 (d, *J* = 7.3 Hz, 1H, ArH); ^13^C NMR (100 MH_Z_, CDCl_3_): δ 23.4, 24.5, 52.7, 98.9, 105.6, 112.6, 118.6, 125.0, 125.2, 126.9, 127.2, 127.9, 129.2, 130.0, 130.2, 131.6, 138.8, 145.0, 150.7, 158.9, 161.3; HRMS (ESI) calculated for C_25_H_22_N_4_, 379.1917 (M+H^+^); found for *m/z*, 379.1916.

**2-Amino-5-(4-chloro-phenyl)-4-piperidin-1-yl-benzo[*h*]quinoline-6-carbonitrile 3b:** Yield:82% (169.0 mg); 0.25 R_f_ (30% ethyl acetate-hexane), golden solid, mp: 127-129 ^o^C; IR (KBr): 3474, 3151, 2929, 2853, 2207 cm^-1^; ^1^H NMR ( 400 MH_Z_ , CDCl_3_): δ 0.66-0.77 (m, 2H, -CH_2_), 1.22-1.48 (m, 4H, -CH_2_-), 2.35-2.45 (m, 2H, -CH_2_-), 2.81-2.91 (m, 2H, -CH_2_-), 4.91 (s, 2H, -NH_2_), 6.29 (s, 1H, ArH), 7.37-7.47 (m, 4H, ArH), 7.62-7.75 (m, 2H, ArH), 8.21 (d, *J* = 8.0 Hz,1H, ArH), 9.08 (d, *J* = 7.3 Hz, 1H, ArH); ^13^C NMR (100 MH_Z_, CDCl_3_): δ 23.3, 24.5, 52.6, 99.1, 105.4, 112.3, 118.4, 125.1, 125.1, 127.1, 127.3, 129.3, 130.0, 131.4, 131.4, 133.8, 137.2, 143.5, 150.7, 158.9, 160.9; HRMS (ESI) calculated for C_25_H_21_ClN_4_O, 413.1527 (M+H^+^); found for *m/z*, 413.1527.

**2-Amino-5-(3-bromo-phenyl)-4-piperidin-1-yl-benzo[*h*]quinoline-6-carbonitrile 3c:** Yield: 65% (149.0 mg); 0.23 R_f_ (30% ethyl acetate-hexane), yellow solid, mp: 204-206 ^o^C; IR (KBr): 3368, 3199, 3058, 2936, 2854, 2210 cm^-1^; ^1^H NMR ( 400 MH_Z_ , CDCl_3_): δ 0.40-0.62 (m, 1H, -CH_2_-), 0.76-0.95 (m, 1H, -CH_2_-), 0.95-1.10 (m, 1H, -CH_2_-), 1.16-1.30 (m, 1H, -CH_2_-), 1.33-1.51 (m, 2H, -CH_2_-), 2.28 (t, *J* = 10.9 Hz, 1H, -CH_2_-), 2.50 (t, *J* = 10.9 Hz, 1H, -CH_2_-), 2.80 (d, *J* = 11.7 Hz, 1H, -CH_2_-), 2.95 (d, *J* = 11.7 Hz, 1H, -CH_2_-), 4.95 (s, 2H, -NH_2_), 6.32 (s, 1H, ArH), 7.29-7.38 (m, 1H, ArH), 7.49-7.60 (m, 3H, ArH), 7.63-7.76 (m, 2H, ArH), 8.23 (d, *J* = 8.0 Hz,1H, ArH), 9.10 (d, *J* = 7.3 Hz, 1H, ArH); ^13^C NMR (100 MH_Z_, CDCl_3_): δ 23.3, 24.4, 51.9, 53.4, 99.6, 105.4, 112.3, 118.3, 121.0, 125.1, 127.2, 128.4, 128.8, 129.2, 130.0, 130.7, 131.3, 133.3, 140.6, 142.9, 150.6, 159.0, 161.0; HRMS (ESI) calculated for C_25_H_21_BrN_4_, 457.1022 (M+H^+^); found for *m/z*, 457.1023.

**2-Amino-5-(4-methoxy-phenyl)-4-piperidin-1-yl-benzo[***h***]quinoline-6-carbonitrile 3d:** Yield: 71% (145.0 mg); 0.14 R_f_ (30% ethyl acetate-hexane), Orange solid; mp: 180-182 ^o^C; IR (KBr): 3437, 3171, 2924, 2852, 2208 cm^-1^; ^1^H NMR ( 400 MH_Z_ , CDCl_3_): δ 0.60-0.80 (m, 2H, -CH_2_-), 1.20-1.46 (m, 4H, -CH_2_-), 2.36 (t, *J* = 10.9 Hz, 2H, -CH_2_-), 2.86 (d, *J* = 11.9 Hz, 2H, -CH_2_-), 3.85 (s, 3H, -O-CH_3_), 4.96 (s, 2H, -NH_2_), 6.23 (s, 1H, ArH), 6.93 (d, *J* = 8.7 Hz, 2H, ArH), 7.42 (d, *J* = 8.7 Hz, 2H, ArH), 7.55-7.74 (m, 2H ,ArH), 8.21 (d, *J* = 7.3 Hz,1H, ArH), 9.09-9.11 (m, 1H, ArH); ^13^C NMR (100 MH_Z_, CDCl_3_): δ 23.4, 24.6, 52.6, 53.3, 98.4, 105.1, 112.3, 112.5, 118.9, 125.0, 125.1, 126.7, 129.1, 129.8, 131.1, 131.5, 131.6, 144.8, 150.7, 158.8, 159.5, 160.9; HRMS (ESI) calculated for C_26_H_24_N_4_O, 409.2023 (M+H^+^); found for *m/z*, 409.2000.

**2-Amino-5-furan-2-yl-4-piperidin-1-yl-benzo[*h*]quinoline-6-carbonitrile 3e:** Yield: 82% (151.0 mg); 0.60 R_f_ (40% ethyl acetate-hexane), brown solid, mp: 212-214 ^o^C; IR (KBr): 3356, 2926, 2856, 2212 cm^-1^; ^1^H NMR ( 400 MH_Z_ , CDCl_3_): δ 0.90-1.25 (m, 4H, -CH_2_-), 1.26-1.45 (m, 2H, -CH_2_-), 2.36 (t, *J* = 10.6 Hz, 2H, -CH_2_-), 2.96 (d, *J*=10.2Hz, 2H, -CH_2_-), 4.87 (s, 2H, -NH_2_), 6.25 (s, 1H, ArH), 6.51-6.58 (m, 1H, ArH), 6.86 (d, *J* = 2.5 Hz, 1H, ArH), 7.46 (s, 1H, ArH), 7.57-7.70 (m, 2H, ArH), 8.17 (d, *J* = 7.3 Hz, 1H, ArH), 9.00 (d, *J* = 8.0 Hz, 1H, ArH); ^13^C (100 MH_Z_, CDCl_3_): δ 23.7, 25.0, 53.1, 99.1, 105.2, 111.0, 111.2, 112.9, 118.1, 125.0, 125.4, 127.4, 129.2, 130.4, 131.3, 133.0, 142.5, 150.3, 150.9, 159.2, 161.5; HRMS (ESI) calculated for C_23_H_20_N_4_O, 369.1710 ; (M+H^+^) found for *m/z*, 369.1689.

**2-Amino-4-piperidin-1-yl-5-thiophen-2-yl-benzo[*h*]quinoline-6-carbonitrile 3f:** Yield: 76% (146.0 mg); 0.64 R_f_ (40% ethyl acetate-hexane), orange solid, mp: 218-220 ^o^C; IR (KBr): 3394, 2925, 2853, 2215 cm^-1^; ^1^H NMR ( 400 MH_Z_ , CDCl_3_): δ 0.80-1.10 (m, 2H, -CH_2_-), 1.21-1.52 (m, 4H, -CH_2_-), 2.37 (t, *J* = 10.9 Hz, 2H, -CH_2_-), 2.96 (d, *J* = 11.7 Hz, 2H, -CH_2_-), 4.95 (s, 2H, -NH_2_), 6.28 (d, *J* = 1.4 Hz, 1H, ArH), 7.11-7.16 (m, 1H, ArH), 7.42-7.47 (m, 1H, ArH), 7.49-7.53 (m, 1H ,ArH), 7.61-7.74 (m, 2H, ArH), 8.21(d, *J* = 8.0Hz, 1H, ArH), 9.06 (d, *J* = 8.0 Hz, 1H, ArH); ^13^C NMR (100 MH_Z_, CDCl_3_): δ 23.4, 24.8, 52.8, 99.4, 106.1, 113.5, 118.3, 125.0, 125.3, 125.6, 127.1, 127.3, 128.0, 129.2, 130.2, 131.4, 137.0, 141.0, 150.3, 159.1, 161.2; HRMS (ESI) calculated for C_23_H_20_N_4_S, 385.1481 (M+H^+^); found for *m/z*, 385.1457.

**2-Amino-5-(2-fluoro-phenyl)-4-piperidin-1-yl-benzo[H]quinoline-6-carbonitrile 3g:** Yield: 60%; 0.21 R_f_ (30% ethylacetate-hexane), grey solid, mp: 187-189^0^C; IR (KBr): 3399, 2938, 2208 cm^-1^; ^1^H NMR ( 400 MH_Z_ , CDCl_3_): δ 0.31-0.46 (m, 1H, -CH_2_-), 0.80-1.06 (m, 2H, -CH_2_-), 1.13-1.27 (m, 1H, -CH_2_-), 1.33-1.50 (m, 2H, -CH_2_-), 2.12-2.27 (m, 1H, -CH_2_-), 2.45-2.57 (m, 1H, -CH_2_-), 2.73-2.85 (m, 1H, -CH_2_-), 2.95-3.06 (m, 1H, -CH_2_-), 4.91 (s, 2H, -NH_2_), 6.35 (s, 1H, ArH), 7.07-7.15 (m, 1H, ArH), 7.25-7.30 (m, 1H, ArH), 7.36-7.45 (m, 1H, ArH), 7.55-7.62 (m, 1H, ArH), 7.63-7.75 (m, 2H, ArH), 8.23 (d, *J* = 7.9 Hz,1H, ArH), 9.12-9.14 (dd, *J* = 1,83 Hz, 1H, ArH); ^13^C NMR (100 MH_Z_, CDCl_3_): δ 23.3, 24.4, 24.7, 52.2, 54.6, 99.8, 106.7, 113.7, 114.9 (d, *J*_C-F_ = 22.0 Hz), 118.1, 123.4, 125.2, 127.2, 127.4, 129.2, 129.8 (d, *J*_C-F_ = 8.6 Hz), 130.3, 131.4, 131.8, 138.4, 150.1, 158.9, 160.3 (d, *J*_C-F_ = 247.2 Hz), 162.0; HRMS (ESI) calculated for C_25_H_21_FN_4_, 397.1823 (MH^+^); found for m/z, 397.1822

**2-Amino-5-(4-chloro-phenyl)-4-morpholin-4-yl-benzo[*h*]quinoline-6-carbonitrile 3h:** Yield: 66% (137.0 mg); 0.21 R_f_ (40% ethyl acetate-hexane), golden solid, mp: 258-260 ^o^C; IR (KBr): 3351, 2924, 2853, 2211 cm^-1^; ^1^H NMR ( 400 MH_Z_ , CDCl_3_): δ 256-2.60 (m, 4H, -CH_2_-), 2.71-2.79 (m, 2H, -CH_2_-), 3.50-3.60 (m, 2H, -CH_2_-), 4.97 (s, 2H, -NH_2_), 6.30 (s, 1H, ArH), 7.36-7.51(m, 4H, ArH), 7.63-7.78(m, 2H, ArH), 8.22 (d, *J* = 8.0 Hz, 1H, ArH), 9.09 (d, *J* = 8.0 Hz, 1H, ArH); ^13^C NMR (100 MH_Z_, CDCl_3_): δ 51.5, 65.6, 99.2, 106.2, 112.0, 118.1, 125.2, 125.3, 127.4, 127.6, 129.6, 130.0, 131.4, 131.4 134.3, 137.4, 142.8, 151.0, 158.9, 159.9; HRMS (ESI) calculated for C_24_H_19_ClN_4_O, 415.1320 (M+H^+^); found for *m/z*, 415.1326.

**2-Amino-5-(4-bromo-phenyl)-4-morpholin-4-yl-benzo[*h*]quinoline-6-carbonitrile 3i:** Yield: 63% (145.0 mg); 0.22 R_f_ (40% ethyl acetate-hexane), orange solid, mp: 260-262^o^C; IR (KBr): 3340, 2925, 2855, 2209 cm^-1^; ^1^H NMR (400 MH_Z_ , CDCl_3_): δ 257-2.68 (m, 4H, -CH_2_-), 2.70-2.76 (m, 2H, -CH_2_-), 3.49-3.59 (m, 2H, -CH_2_-), 5.00 (s, 2H, -NH_2_), 6.30 (s, 1H, ArH), 7.34-7.40 (m, 2H, ArH), 7.56-7.62 (m, 2H, ArH), 7.64-7.77(m, 2H, ArH), 8.22 (d, *J* = 7.3 Hz,1H, ArH), 9.07-9.12 (dd, *J* = 0.9 Hz, 1H, ArH); ^13^C NMR (100 MH_Z_, CDCl_3_): δ 51.5, 65.6, 99.3, 106.1, 112.0, 118.1, 122.4, 125.1, 125.3, 127.4, 129.6, 130.0, 130.6, 131.4, 131.6, 137.9, 142.8, 150.8, 158.9, 159.9; HRMS (ESI) calculated for C_24_H_19_BrN_4_O, 459.0815 (M+H^+^); found for *m/z*, 459.0816.

**2-Amino-5-(4-methoxy-phenyl)-4-morpholin-4-yl-benzo[*h*]quinoline-6-carbonitrile 3j:** Yield 68% (140.0 mg); 0.15 R_f_ (40% ethyl acetate-hexane), orange solid, mp: 278-280 ^o^C; IR (KBr): 3357, 2925, 2854, 2209 cm^-1^; ^1^H NMR ( 400 MH_Z_ , CDCl_3_): δ 2.55-2.72 (m, 4H, -CH_2_-), 2.77-2.83 (m, 2H, -CH_2_-), 3.46-3.58 (m, 2H, -CH_2_-), 3.87 (s, 3H, -O-CH_3_), 4.94 (s, 2H, -NH_2_), 6.27 (s, 1H, ArH), 6.95-7.00 (m, 2H, ArH), 7.39-7.47 (m, 2H, ArH), 7.361-7.75 (m, 2H, ArH), 8.22 (d, *J* = 8.0 Hz,1H, Ar-H), 9.06-9.11 (m, 1H, Ar-H); ^13^C (100 MH_Z_, CDCl_3_): δ 51.5, 55.3, 65.7, 98.6, 106.0, 112.0, 112.8, 118.6, 125.1, 125.2, 127.0, 129.4, 129.8, 131.2, 131.5, 131.6, 144.1, 150.8, 158.7, 159.8, 160.0; HRMS (ESI) calculated for C_25_H_22_N_4_O_2_, 411.1816 (M+H^+^); found for *m/z*, 411.1816.

**Molecular docking:** Molecular modeling studies of benzo[*h*]quinoline derivatives were carried out using the molecular modeling software Sybyl-X 2.0 (Tripos International).Drawing of structures and simple geometry optimisation were performed withChem Bio-Office suite Ultra v12.0 (2012) (Cambridge Soft Corp).The binding affinity of all compounds was predicted with the cancer target enzyme cyclin-dependent kinase 2 (CDK2). The Surflex-Dock module in Sybyl was used to construct a 3D model of the structures. Energy minimization was done using the Tripos force field witha distance-dependent dielectric and the Powell gradient minimization algorithm, with a convergence criterion of 0.001 kcal mol^−1^ for the determination of conformations with the most favourable (lowest energy). Many X-ray diffraction crystalstructures of human cancer protein targets are available in the Protein Data Bank (PDB; http://www.rcsb.org).Aromatase at 3.5Å resolution (PDB: 3EQM^35,36^ and CDK2 at a1.65 Å resolution (PDB ID: 2R3J)^37,38^ were selected. The crystal structure of CDK2 in complex with 3-bromo-5-phenyl-N-(pyridin-3-ylmethyl)pyrazolo[1,5-*a*]pyrimidin-7-amineand the structure of thealpha/beta-tubulin dimer in complex with taxolwere obtained from the PDB. Hydrogen atoms were added to the protein with the protonation 3D toolin Sybyl. Partial atomic charges were assignedusing theGasteiger-Hückel method in Sybyl. All 2D structures were converted to 3D structures using the program Concord v4.0 and the maximum number of iterations performed in the energy minimization was set to 2000. Further geometry optimization was done with theMOPAC-6 package using the semi-empirical PM3 Hamiltonian method^39-47^.

**Prediction of*in silico* pharmacokinetic and toxicity parameters:** Pharmacokinetic (PK) properties depend on the chemical propertiesof drugs,which determine their absorption, distribution, metabolism, excretion, and toxicity (ADMET) properties, which are the key descriptors for the human therapeutic use of any compound.Predictive ADMET mathematical models were derivedwithdifferent PKparameters, namely, aqueous solubility, blood-brain barrier penetration, cytochrome P-450 2D6 inhibition, hepatotoxicity, human intestinal absorption and plasma protein binding. Predictions from these models were contrasted withknownrules for appropriate ADMET characteristics for all benzo[*h*]quinoline derivatives. Some properties correlate well with PK,e.g, primary determinant of fractional absorption can be representedby polar surface area (PSA) (cut-off ≤140Å^2^) and low molecular weight (MW) for absorption^39-41^. For secondary determination of fractional absorption (passive membrane transport), the sum of H-bond donors and acceptors (cut-off ≤12) was used. The number of rotatable bonds is used as a measure of flexibility (cut-off ≤10) and bioavailability. Drug distribution depends on a number of factors,such as permeability (indicated by apparent Caco-2 and MDCK permeability, logKp for skin permeability), blood-brain barrier (log BB), the volume of distribution and plasma protein binding (logKhsa for serum protein binding)^42-45^. These ADME descriptors were calculated and compared with standard ranges. The octanol-water partition coefficient (logP) has been implicated in BB penetration and permeability studies. Excretion of drugs from the body depends on logP and MW. Likewise, rapid renal clearance is associated with hydrophilicity and small MW. In the liver, drug metabolism is associated with hydrophobicity and large MW. Higher lipophilicity leads to poor absorption and increase in metabolic processes. ADMEdescriptors for90% of orally active compounds follow Lipinski’s rule of 5. These ADME parameters were calculated through Qikprop v3.2 (Schrödinger, LLC, USA, 2015 and Discovery Studio 3.5).The recommended toxicity screening models for carcinogenicity are developmental toxicity, mutagenicity and skin irritancy or sensitization,and these were calculated with the DSTOPKAT module. These predictions are useful for the optimization of therapeutic ratios of lead compounds and assessment of their potential safety^40-45^. These predictions also help in evaluating intermediates, metabolites and pollutants, along with setting dose range for animal assays.

**Supplementary Table S1.**Predicted ADME parameters of active benzo[*h*]quinoline derivatives.

| Compound | log S(aqueous solubility) | log Khsa(serum protein binding) | log BB(brain/blood) | No. of metabolic reactions | Predicted CNS activity | log HERG (K+ channel blockage) | Apparent  Caco-2 permeability (nm/sec) | Apparent  MDCK permeability(nm/sec) | log Kp (skin  permeability) | % human oral absorption in GI (+-20%) | vdW polar SA (PSA) | Qualitativemodel for human oral absorption |
| --- | --- | --- | --- | --- | --- | --- | --- | --- | --- | --- | --- | --- |
| 3a | -6.176 | 0.743 | -0.721 | 0 | -1 | -5.149 | 632.794 | 301.674 | -2.407 | 100 | 61.144 | High |
| 3b | -6.739 | 0.855 | -0.576 | 0 | 0 | -4.995 | 632.794 | 658.361 | -2.551 | 100 | 61.144 | Low |
| 3c | -6.91 | 0.908 | -0.574 | 0 | 0 | -5.063 | 632.771 | 686.047 | -2.532 | 100 | 61.145 | Low |
| 3d | -6.217 | 0.783 | -0.771 | 1 | -1 | -4.858 | 632.823 | 301.689 | -2.479 | 100 | 69.535 | Low |
| 3e | -5.884 | 0.584 | -0.659 | 1 | 0 | -4.948 | 624.068 | 297.18 | -2.632 | 100 | 68.95 | High |
| 3f | -6.19 | 0.749 | -0.567 | 1 | 0 | -4.707 | 626.59 | 413.049 | -2.671 | 100 | 61.129 | High |
| 3g | -6.37 | 0.767 | -0.655 | 0 | 0 | -5.056 | 636.228 | 428.3 | -2.488 | 100 | 61.143 | Low |
| 3h | -6.172 | 0.516 | -0.539 | 1 | 0 | -5.102 | 701.273 | 752.471 | -2.429 | 100 | 67.722 | High |
| 3i | -6.269 | 0.532 | -0.536 | 1 | 0 | -5.153 | 702.405 | 793.41 | -2.427 | 100 | 67.855 | Low |
| 3j | -5.732 | 0.429 | -0.773 | 2 | -1 | -5.111 | 680.834 | 326.503 | -2.38 | 100 | 75.464 | High |
| Dox | -2.269 | -0.604 | -2.71 | 9 | -2 | -5.908 | 3.532 | 1.224 | -7.566 | 0 | 203.041 | Low |
| **Standard Range** | (-6.5 / 0.5) | (-1.5 / 1.5) | (-3.0 / 1.2) | (1.0 / 8.0) | –2 (inactive)  +2 active) | (concern below -5) | (<25 poor, >500 great) | (<25 poor, >500 great) | (–8.0 to –1.0, Kp in cm/hr) | (<25% is poor) | ( 7.0 / 200.0) | (>80% is high) |

For 95% of known drugs based on predictions usingQikprop v3.2 (Schrödinger, 2011).


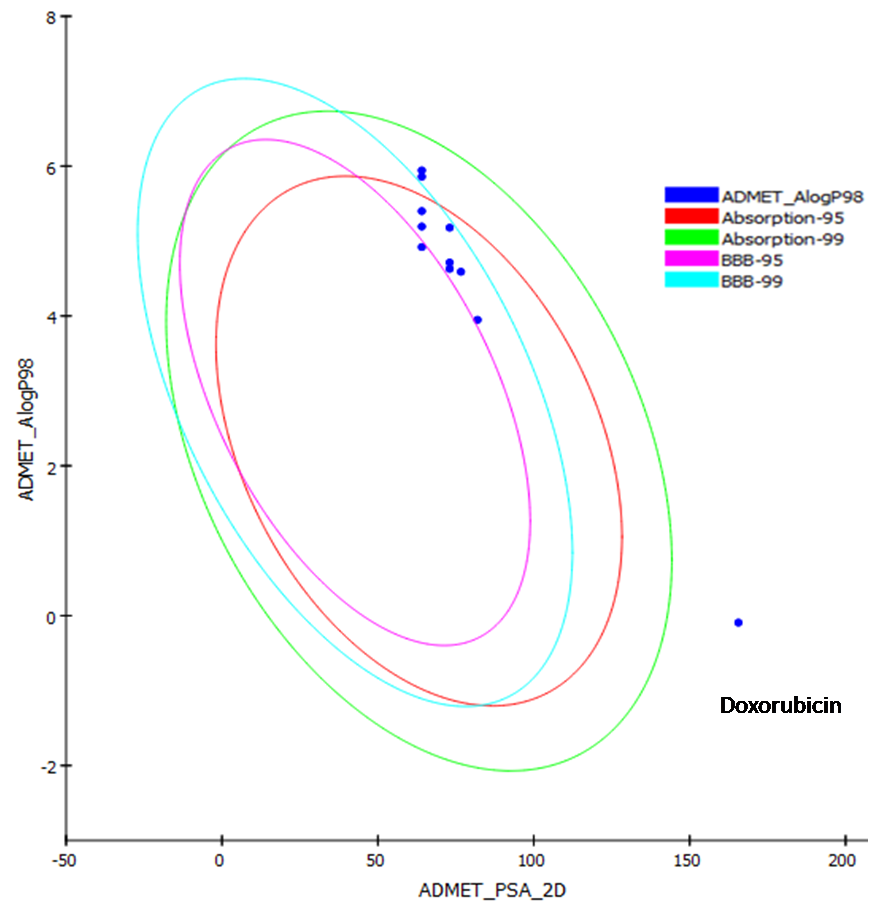


**Supplementary Figure S1.** Plot of polar surface area (PSA) versus ALogP for benzo[*h*]quinoline derivatives. The 95% and 99% confidence limit ellipses corresponding to the blood brain barrier (BBB) and intestinal absorption are shown separately.

**Spectra of reported compounds**


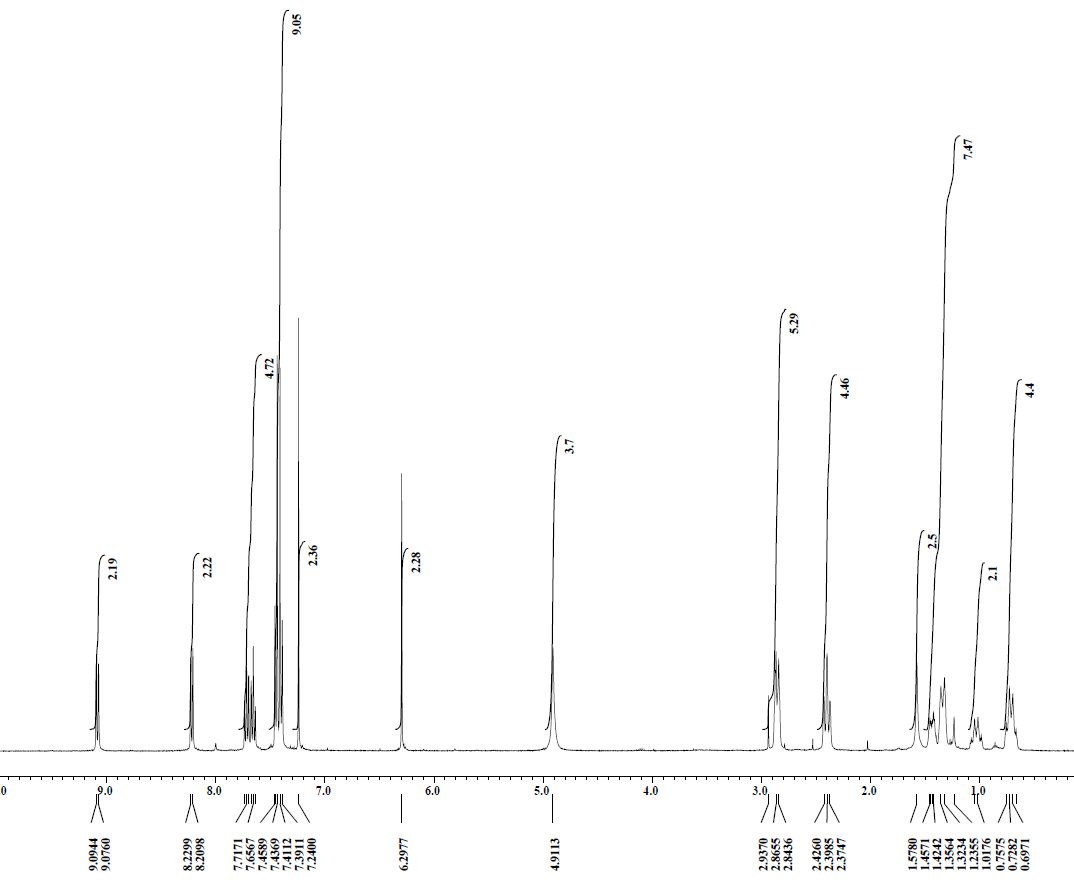


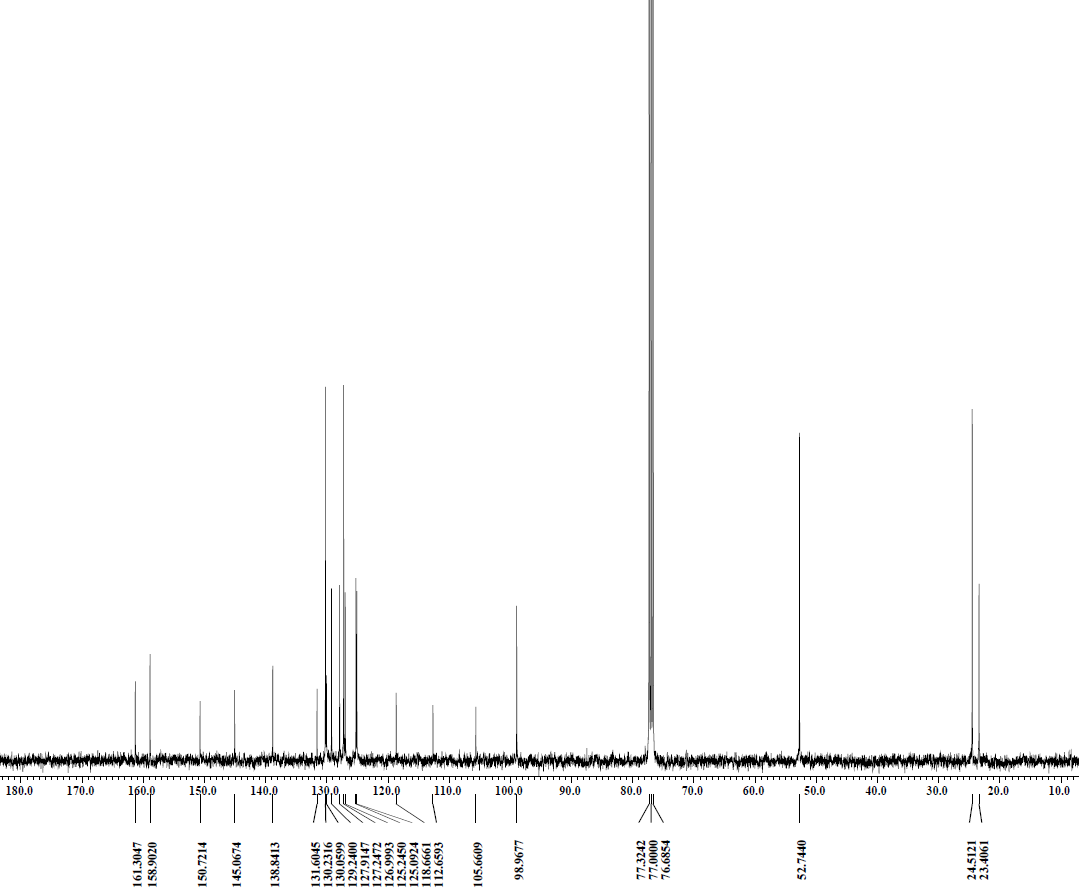


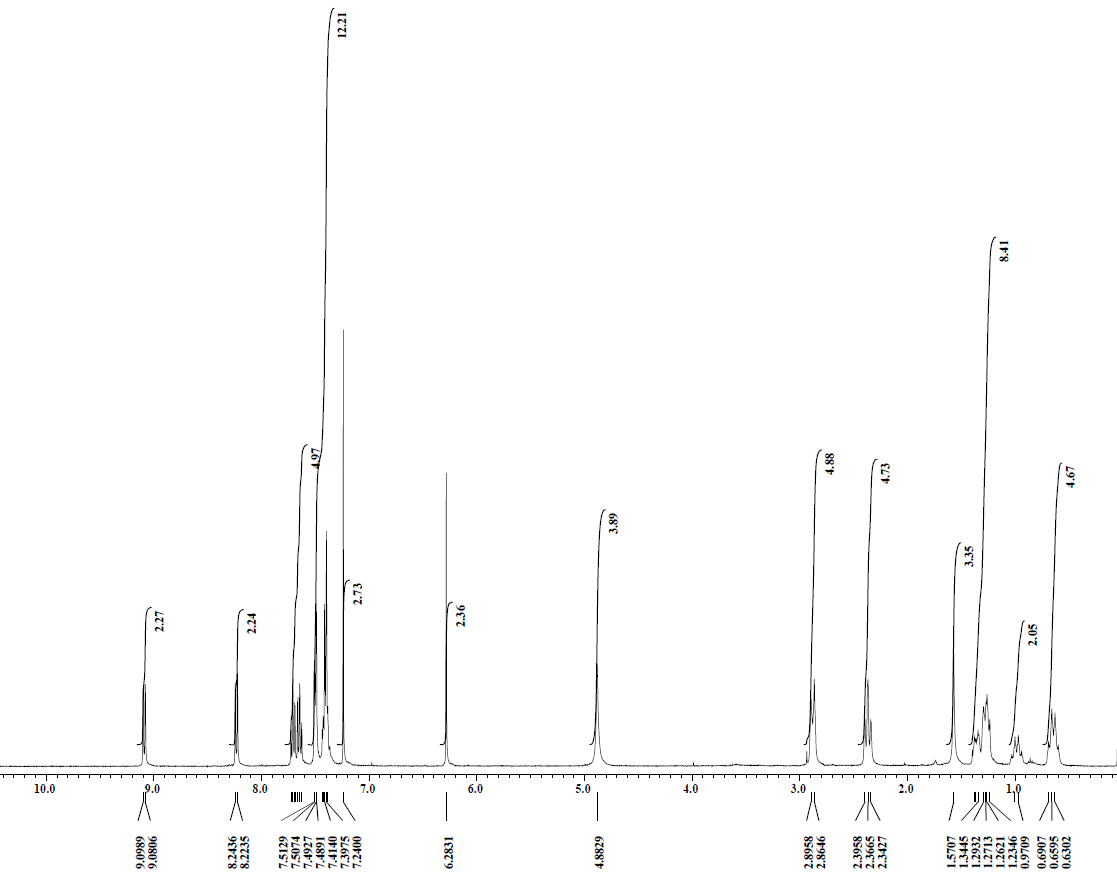


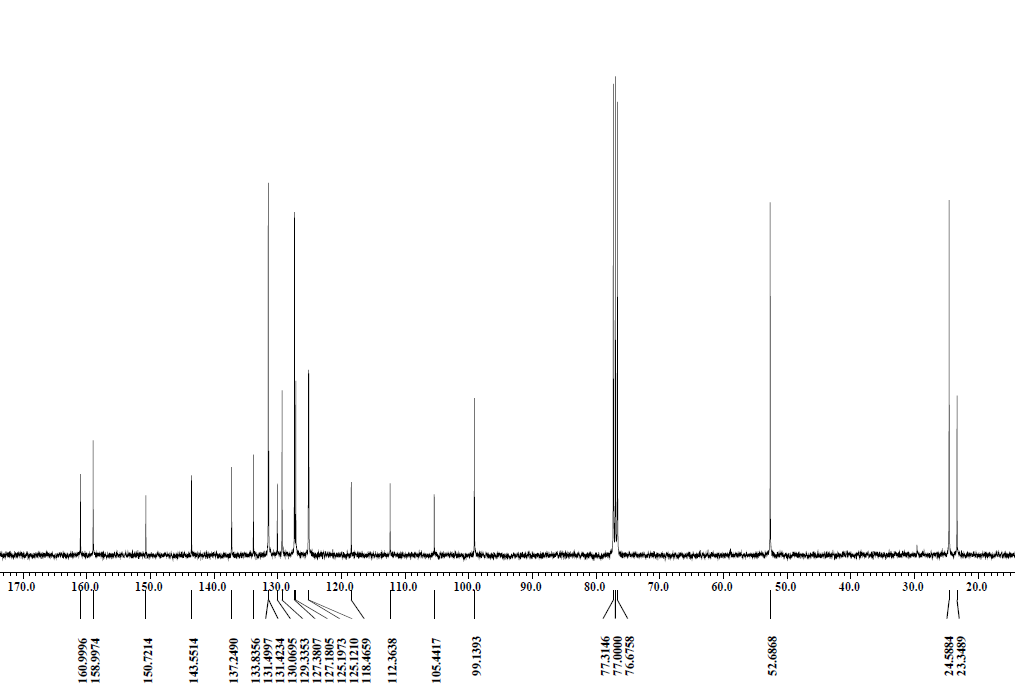


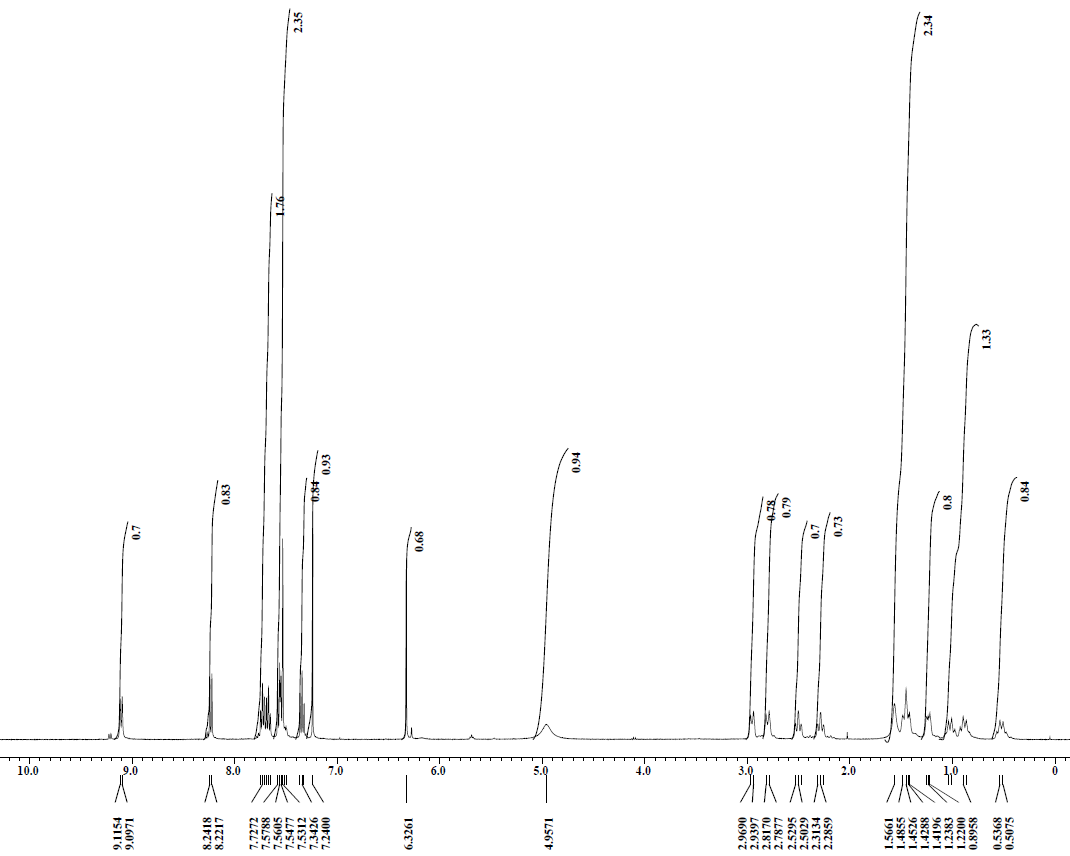


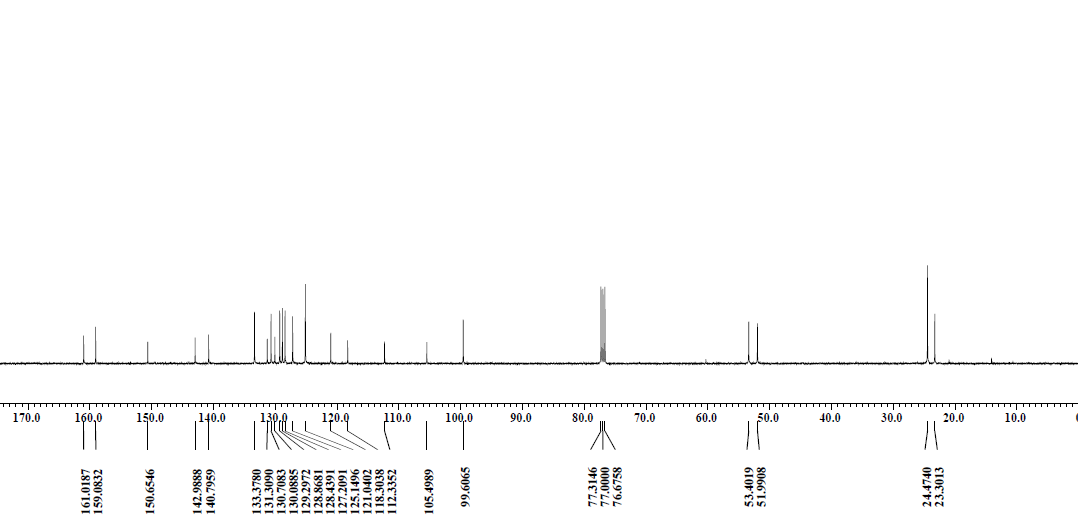


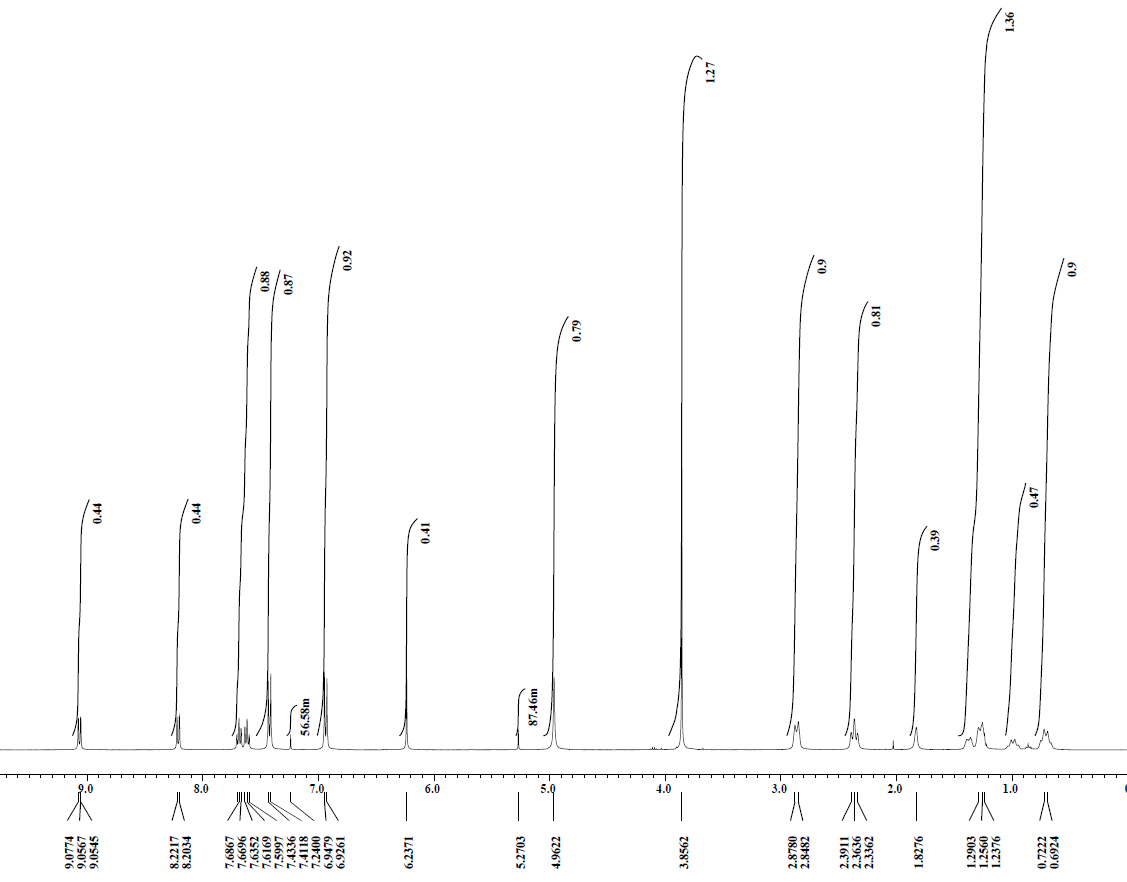


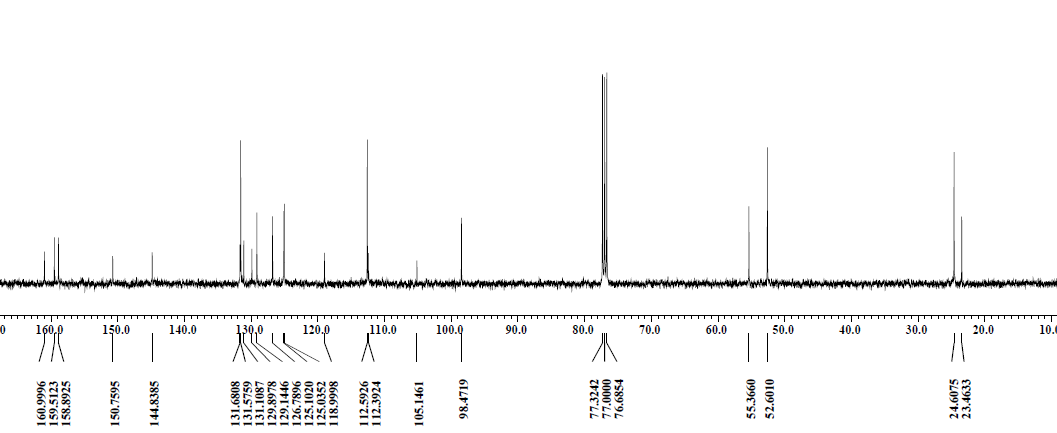


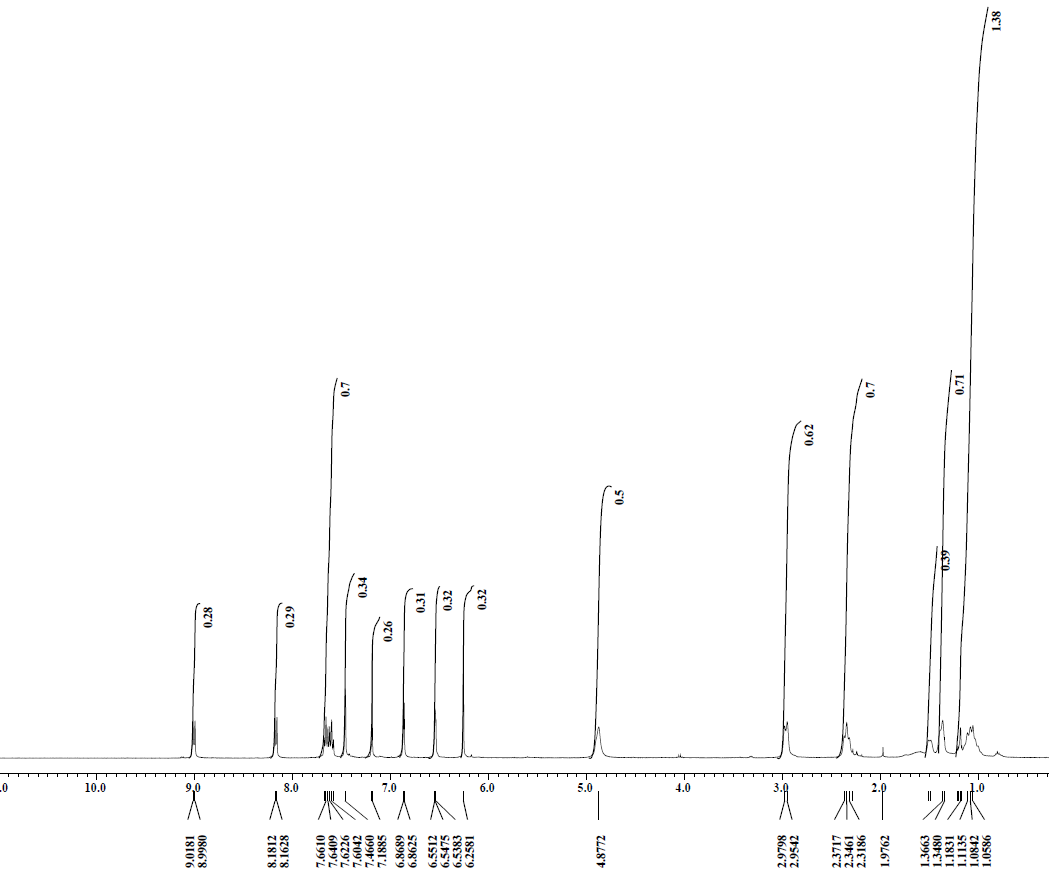


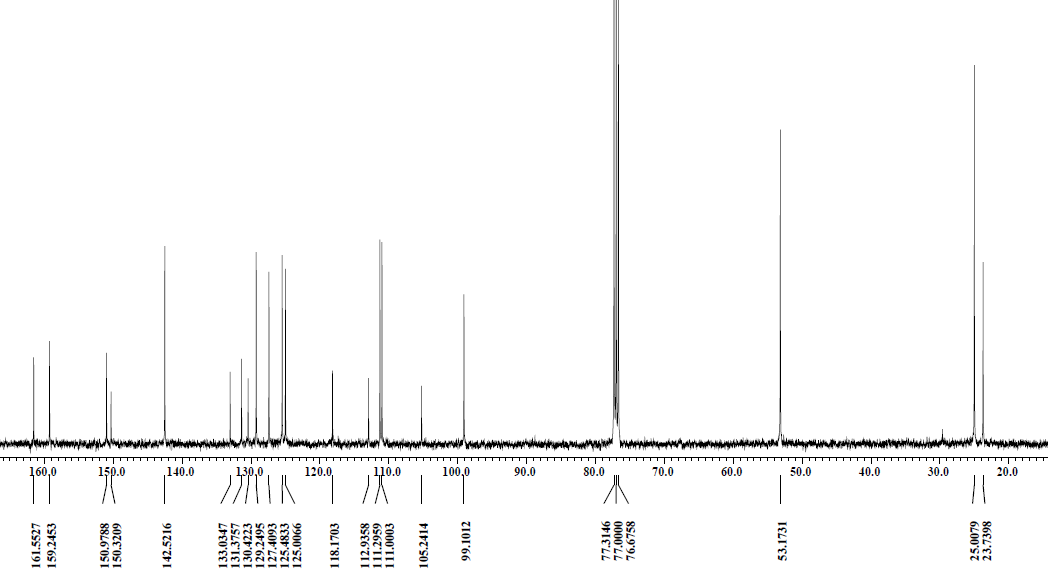


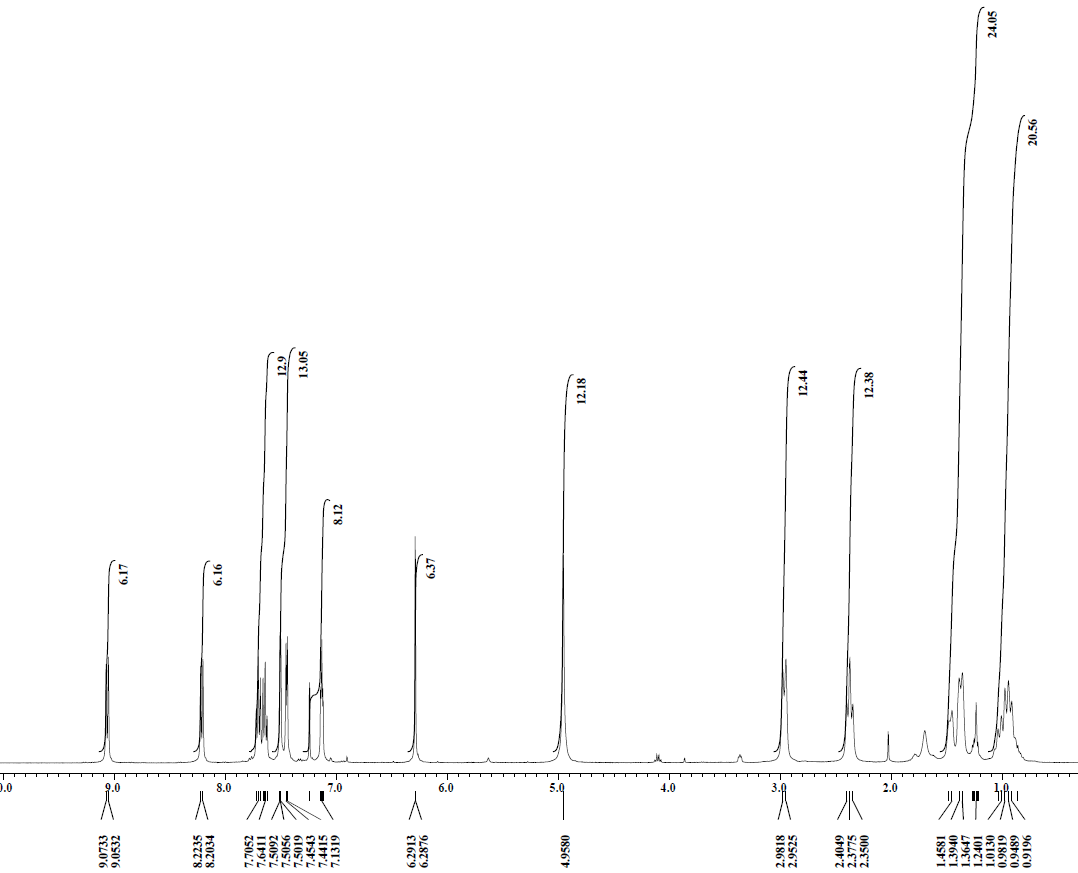


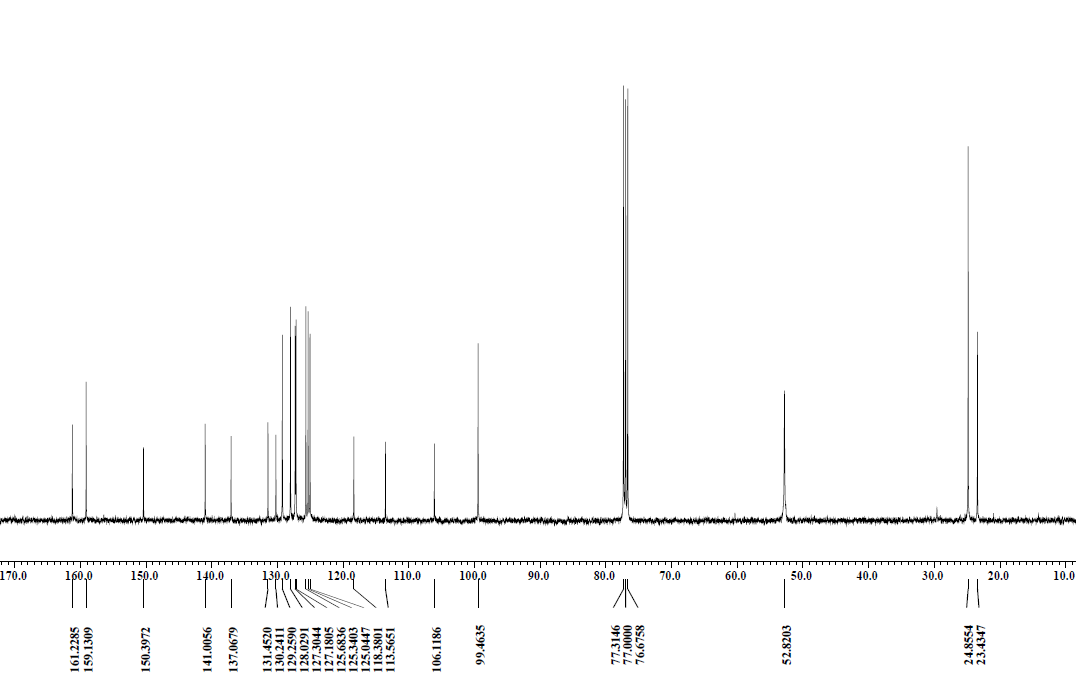


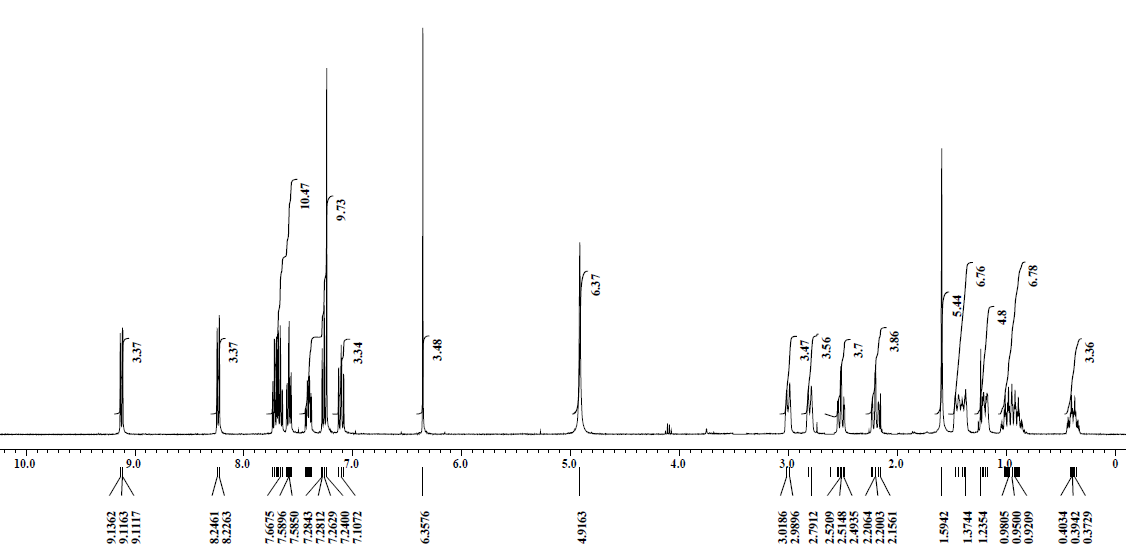


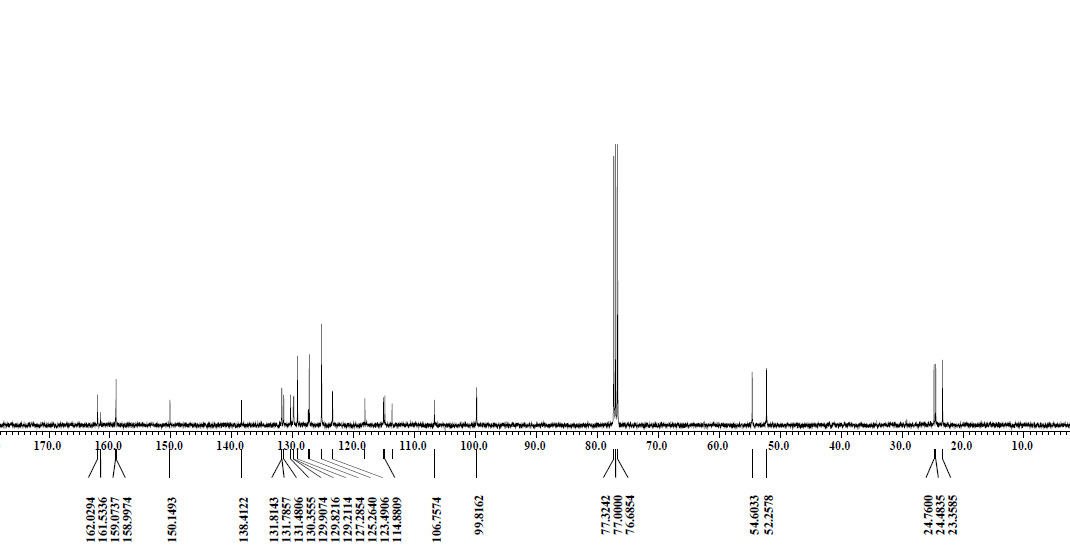


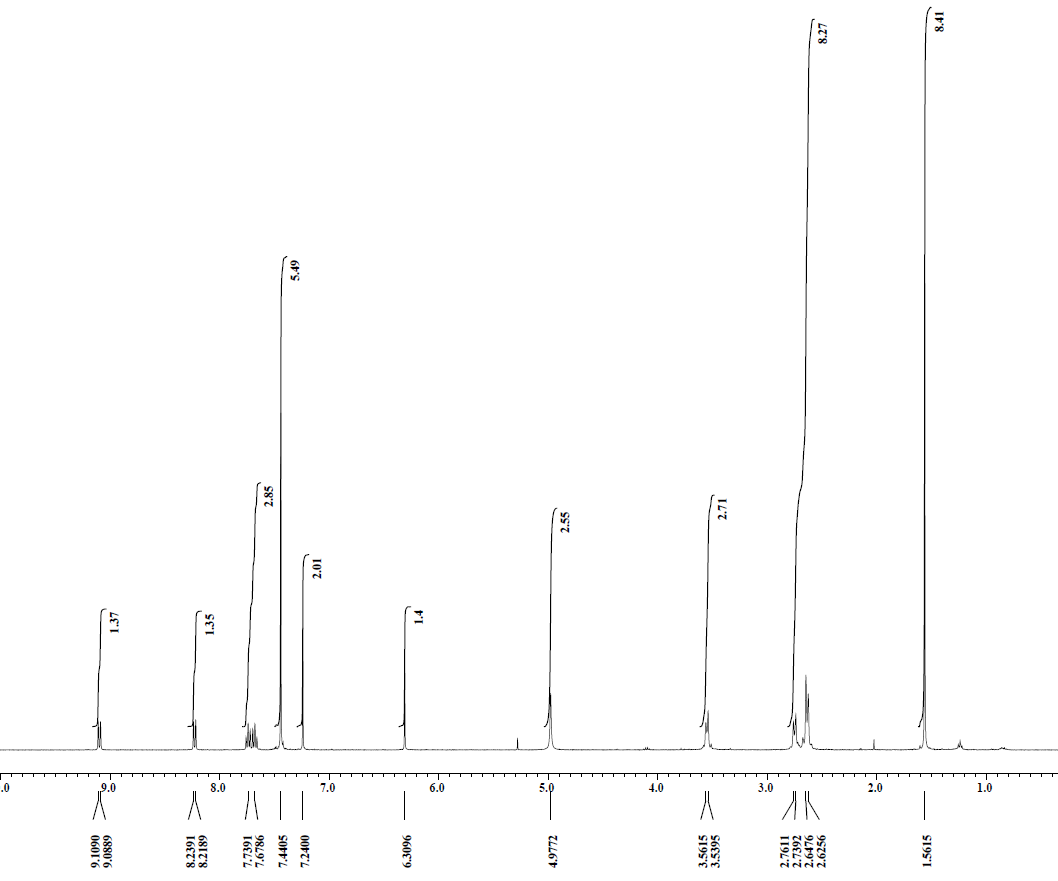


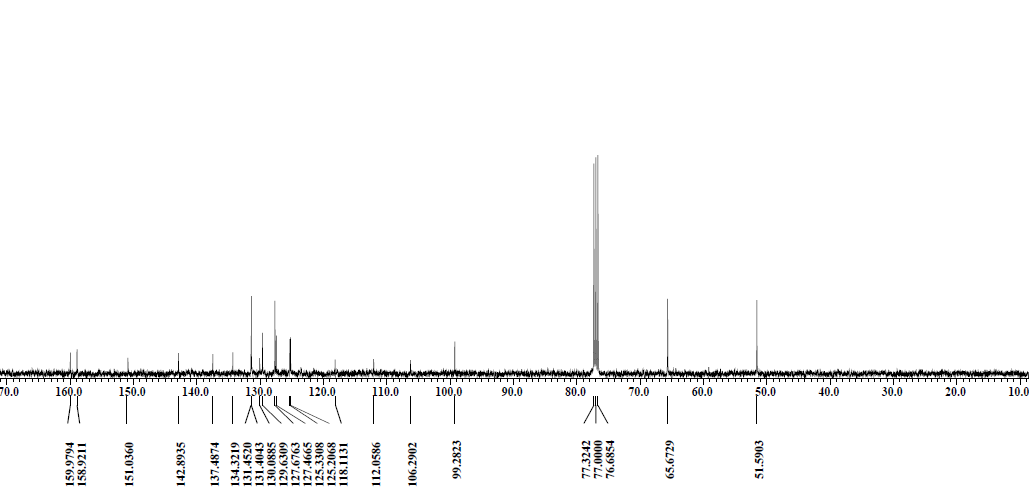


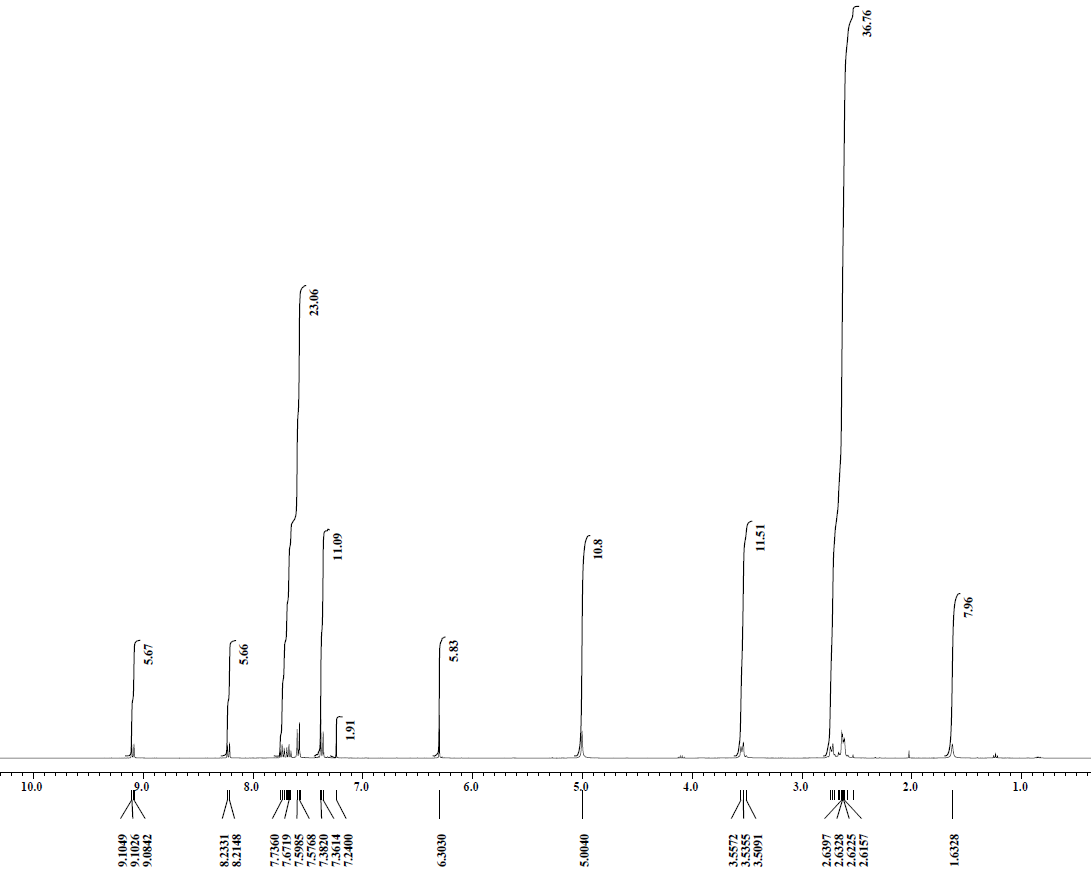


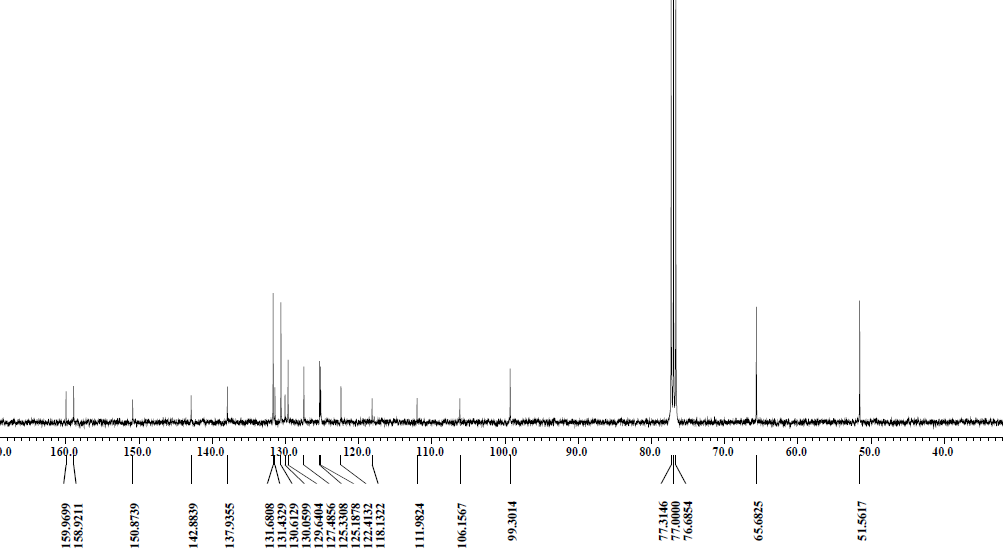


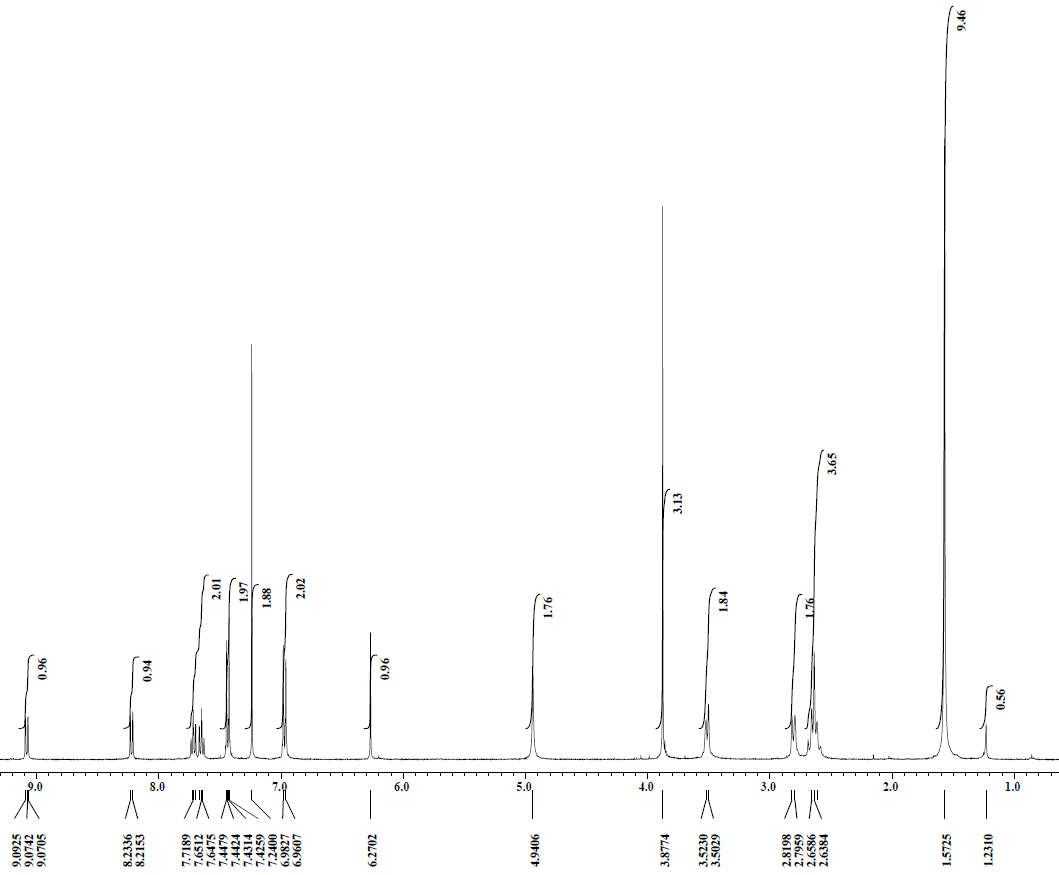


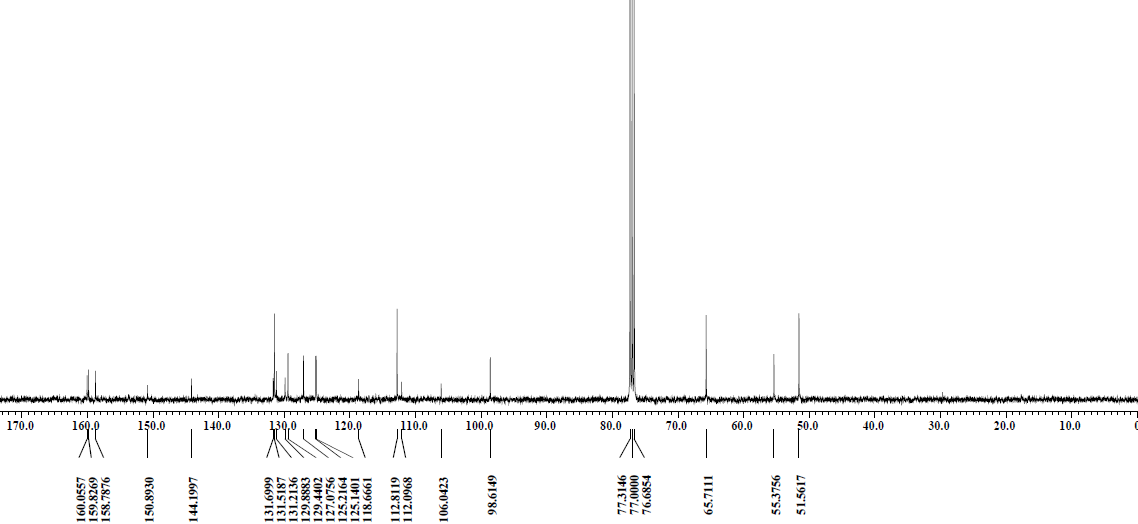

Supplement: Supplementary Information [file srep38128-s1.doc]
